# Synthesis and Optical Properties of Diboron Complexes of 1,4‐Anthraquinone Derivatives

**DOI:** 10.1002/chem.71179

**Published:** 2026-05-25

**Authors:** Yasuhiro Kubota, Ayumi Ogasawara, Ayato Hakamada, Naoya Suzuki, Masayasu Taki, Shota Mizuno, Toshiyasu Inuzuka, Kazumasa Funabiki, Chie Yamada‐Kubota, Miho Kuwahara, Tetsuya Hara

**Affiliations:** ^1^ Department of Chemistry and Biomolecular Science Faculty of Engineering Gifu University Gifu Japan; ^2^ Department of Chemistry Graduate School of Science Nagoya University Nagoya Japan; ^3^ Institute For Glyco‐core Research (iGCORE) Gifu University Gifu Japan; ^4^ Life Science Research Center Gifu University Gifu Japan; ^5^ Department of Occlusal and Oral Functional Rehabilitation Graduate School of Medicine Dentistry, and Pharmaceutical Sciences Okayama University Okayama Japan

**Keywords:** anthraquinone, boron complex, near infrared fluorescence, photoinduced electron transfer, *β*‐Iminoenolate structure

## Abstract

N^O‐type 1,4‐diboron complexes (**5**: R = H, **6**: R = CF_3_, **7**: R = OMe, and **8**: R = NMe_2_) were synthesized via a simple two‐step reaction and their photophysical properties were systematically investigated. Single‐crystal x‐ray diffraction analysis of **5** revealed a planar diboron chromophore with two *β*‐iminoenolate structures, while the *N*‐phenyl substituents are significantly twisted from the chromophore plane. Boron complexation rigidified the chromophore, affording vibronically resolved and spectrally narrowed absorption bands for **5**–**7**, whereas **8** exhibited pronounced spectral broadening accompanied by a bathochromic shift. TDDFT calculations indicated that increasing electron‐donating ability shifts the S_0_–S_2_ transition to longer wavelengths, and in **8** this transition overlaps with S_0_–S_1_, accounting for the spectral broadening. Complexes **5**–**7** exhibited near‐infrared fluorescence, while **8** was nonfluorescent. Dynamic quenching of **5** by *N*,*N*‐dimethylaniline via intermolecular photoinduced electron transfer (PeT) was confirmed by Stern–Volmer analysis with lifetime shortening. The decrease in fluorescence quantum yield with increasing electron‐donating ability was attributed to intramolecular PeT, and protonation restored fluorescence. The nonfluorescent nature of the 1,5‐diboron complex was attributed to enhanced nonradiative decay in accordance with the energy gap law, together with possible intramolecular PeT.

## Graphical abstract

1,4‐Diboron complexes exhibit near‐infrared fluorescence, in contrast to non‐emissive 1,5‐diboron analogues. Fluorescence quantum yields decrease with increasing electron‐donating substituents, attributed to enhanced nonradiative decay via intramolecular PeT. The non‐emissive nature of 1,5‐diboron complexes is suggested to arise from efficient intramolecular PeT and energy gap law effects associated with red‐shifted absorption.

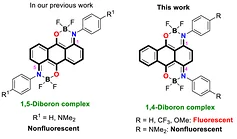

## 1 Introduction

Organoboron complexes represent a prominent class of fluorescent dyes [1, 2, 3, 4, 5, 6]. Among them, BODIPY (4,4‐difluoro‐4‐bora‐3a,4a‐diaza‐*s*‐indacene) dye is the most well‐known and has been the subject of numerous comprehensive reviews [7, 8, 9, 10, 11, 12]. BODIPY dyes comprise dipyrromethene cores rigidified via a nitrogen–boron–nitrogen (N–B–N) bridging motif. This structural rigidification suppresses non‐radiative decay pathways, thereby enhancing the fluorescence quantum yield (*Φ*_f_) of the ligand dye. Moreover, the photophysical properties of organoboron complexes are highly dependent on the structure of the dye ligand. Therefore, boron complexation of organic dyes offers an effective strategy for the development of novel fluorescent materials. Indeed, organoboron complexes have been extensively explored for applications across diverse fields, including optoelectronics [13, 14, 15, 16] and biomedicine [17, 18, 19, 20]. The design and synthesis of new boron‐based complexes remain an area of active research [21, 22, 23, 24, 25, 26, 27, 28].

**FIGURE 1:**
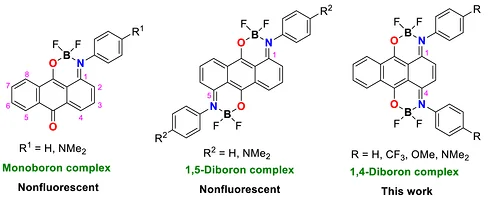
N^O‐type boron complexes of anthraquinone.

On the other hand, natural anthraquinones were historically employed as the principal dyes in textile applications [29, 30]. Even today, anthraquinone derivatives remain widely applied in various colorant applications, including textiles and food products [31, 32]. In addition, anthraquinones are well known for their remarkable anticancer properties, leading to the design and synthesis of numerous novel derivatives with a wide range of biological activities [33]. Anthraquinone derivatives are widely employed in various applications such as colorimetric sensors [34, 35], non‐linear optical (NLO) materials [36], liquid crystals [37, 38] and hydrogen peroxide production [39, 40], owing to their exceptional thermal and photochemical stability, coordination ability, electron‐accepting properties, rigid molecular framework, and reversible redox behavior. Some substituted anthraquinones exhibit fluorescence. Anthraquinones possessing a low‐energy π,π* state are particularly prone to fluorescence [41]. Anthraquinones displaying solvatochromic fluorescence [42, 43], aggregation‐induced emission (AIE) [44, 45], mechanochromic luminescence (MCL) [46, 47], and thermally activated delayed fluorescence (TADF) [48, 49] have been reported. Owing to these fluorescence properties, anthraquinones have been applied in image‐guided photodynamic therapy [50], intracellular reactions [51], fluorescent imaging [52], and organic light‐emitting diodes (OLEDs) [53]. There have been only a few reports on boron complexes of anthraquinone. Although the synthesis of O^O‐type boron complexes of anthraquinone has been reported by P. N. Preston et al. [54, 55] and H. Hartmann et al. [56], and that of N^O‐type boron complexes by M. V. Gorelik et al. [57], their optical properties are not reported in the literature. For O^O‐type boron complexes of anthraquinone, K. Ono et al. reported that naphthacenequinone‐based complexes exhibit fluorescence [58, 59]. In the context of N^O‐type boron complexes of anthraquinone, we previously reported that monoboron and 1,5‐diboron complexes exhibit near‐infrared panchromatic absorption without detectable fluorescence (Figure 1) [60]. In this study, we report the synthesis and photophysical properties of 1,4‐diboron complexes derived from 1,4‐bis(arylamino)anthraquinone (Figures 1, R = H, CF_3_, OMe, NMe_2_), demonstrating that, in contrast to the 1,5‐analogues, these complexes exhibit near‐infrared fluorescence. The fluorescence quantum yields were found to decrease with increasing electron‐donating ability of the substituent R. Mechanistic investigations revealed that this quenching predominantly arises from intramolecular photoinduced electron transfer (PeT), as supported by intermolecular PeT quenching experiments upon addition of dimethylaminobenzene (DMA) to 1,4‐diboron complex **5** (R = H). Furthermore, DFT calculations suggest that intramolecular PeT is more favorable in 1,5‐diboron complexes than in the 1,4 counterparts, thereby providing new insight into the structure–property relationship governing emissive versus non‐emissive behavior in diboron complexes of anthraquinone.

## 2 Synthesis

**SCHEME 1:**
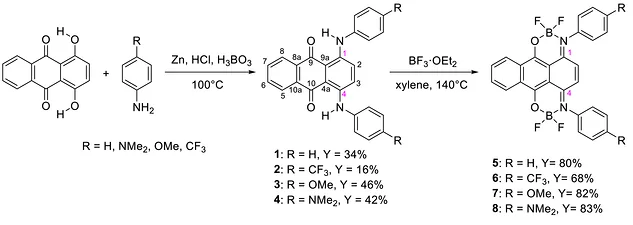
Synthesis of diboron complexes **5**−**8** derived from 1,4 bis(arylamino)anthraquinone.

1,4‐Bis(arylamino)anthraquinones **1** [61], **3** [62], and **4** [63] are known compounds. Compounds **1**–**4** were synthesized by the reaction of 1,4‐dihydroxyanthraquinone with various aniline derivatives, as shown in Scheme 1. In this reaction, 1,4‐dihydroxyanthraquinone was first reduced with hydrogen to yield leucoquinizarin, which subsequently reacted with aniline derivatives in the presence of boric acid to afford the corresponding 1,4‐bis(arylamino)anthraquinones. The resulting anthraquinones **1**–**4** were then treated with boron trifluoride–diethyl ether complex to yield the corresponding boron complexes **5**–**8** (Scheme 1). In the ^1^ ^3^C NMR spectra of compounds **1**–**4** and **6**–**8** recorded in CDCl_3_, the two aryl groups on the nitrogen atoms exhibited non‐equivalent carbon signals, indicating restricted rotation or asymmetry around the nitrogen centers.

## 3 Crystal Structures

**FIGURE 2:**
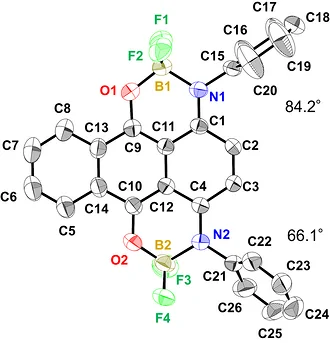
ORTEP drawing of **5**. Hydrogen atoms are omitted for clarity. The dihedral angles between the anthraquinone moiety and the phenyl ring are shown.

Single crystal of **5** was obtained by slow diffusion of *n*‐hexane into a solution of **5** in dichloromethane. The x‐ray crystallographic structures of **5** is shown in Figure 2. The chromophore of **5**, namely, the anthraquinone–diboron moiety, is highly planar and adopts an asymmetric structure in the crystalline state. The dihedral angles between the anthraquinone–diboron moiety and the two phenyl groups are 84.2° and 66.1°, respectively. Experimentally obtained bond lengths of **5** in the crystalline state are shown in Figure S1. The C1─C11 and C4─C12 bond lengths (both 1.43 Å) are longer than those of C9─C11 (1.38 Å) and C10─C12 (1.39 Å). The C1─N1 (1.32 Å) and C4─N2 (1.33 Å) bond lengths are more similar to that of a typical C═N double bond (ca. 1.29 Å) than to that of a C─N single bond (ca. 1.47 Å) [64]. Additionally, the C9─O1 and C10─O2 bond lengths (both 1.32 Å) are intermediate between those of a typical single C─O bond (ca. 1.42 Å) and a typical double C═O bond (ca. 1.22 Å) [64]. These results suggest that the bidentate ligand of **5** adopts a delocalized *β*‐iminoenolate structure rather than the *β*‐ketoiminate structure in the crystalline state (Figure S2).

## 4 UV−Vis−NIR Absorption Properties

**FIGURE 3:**
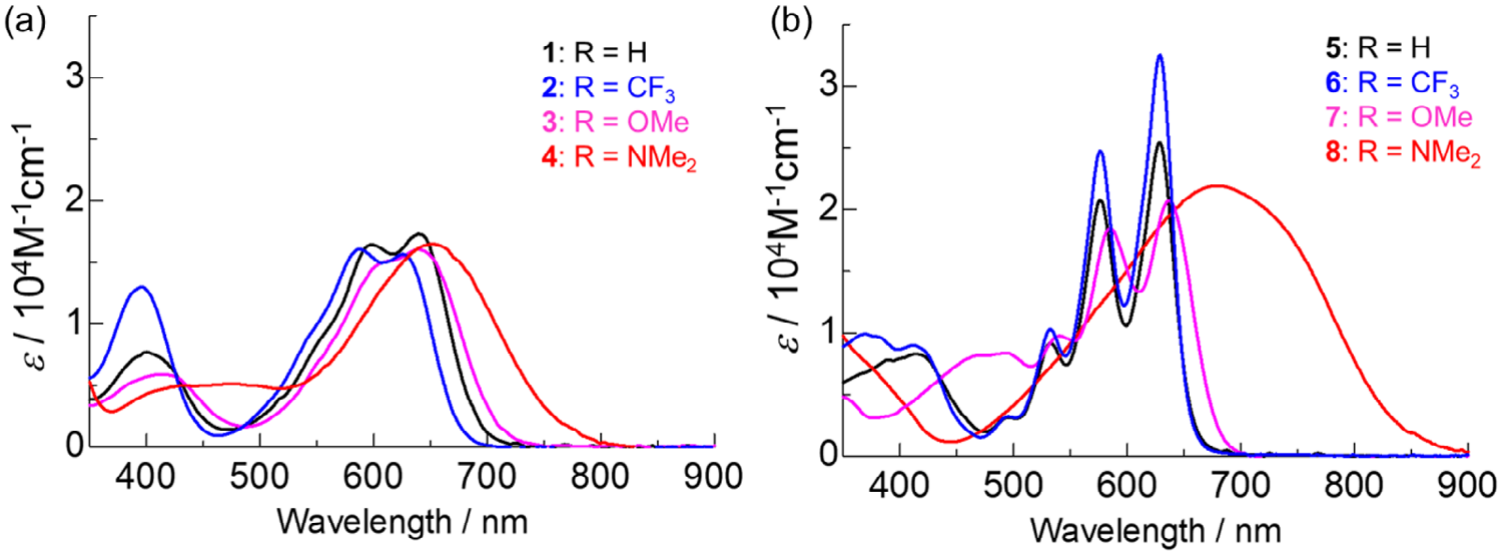
UV−vis−NIR absorption spectra of (a) 1,4‐bis(arylamino)anthraquinones **1**−**4** and (b) 1,4‐diboron complexes **5**−**8** in dichloromethane.

The UV−Vis−NIR absorption spectra of 1,4‐bis(arylamino)anthraquinones **1**−**4** and 1,4‐diboron complexes **5**−**8** in dichloromethane are shown in Figure 3. The maximum absorption wavelengths (*λ*_max_), molar extinction coefficients (*ε*), and full widths at half‐maximum (fwhm) are summarized in Table 1. Boron complexation rigidifies the chromophore, leading to distinct vibrational peaks in the absorption spectra of complexes **5**−**7**. For example, in the spectrum of **5**, the peaks at 628, 576, 532, and 496 nm are assigned to the S_0,0_−S_1,0_, S_0,0_−S_1,1_, S_0,0_−S_1,2_, and S_0,0_−S_1,3_ transitions, respectively.

**TABLE 1:** UV‐vis‐NIR absorption properties in dichloromethane.

Compd	*λ*_max_/nm (*ε*)	fwhm^a^ (cm^−1^)
**1**	400 (7,700), 598 (16,400), 640 (17,300)	3,140
**2**	396 (13,000), 587 (16,100), 625 (15,700)	3,310
**3**	410(5,900), 639 (16,000)	3,170
**4**	477 (5,100), 651 (16,500)	3,440
**5**	414 (8,300), 496 (3,100), 532 (9,200), 576 (20,800), 628 (25,400)	2,280
**6**	412 (9,000), 496 (3,200), 532 (10,300), 576 (24,700), 628 (32,500)	2,180
**7**	493 (8,400), 540 (9,800), 585 (18,400), 637 (20,700)	2,660
**8**	680 (21,900)	4,900

^a^ Full width at half‐maximum height.

**FIGURE 4:**
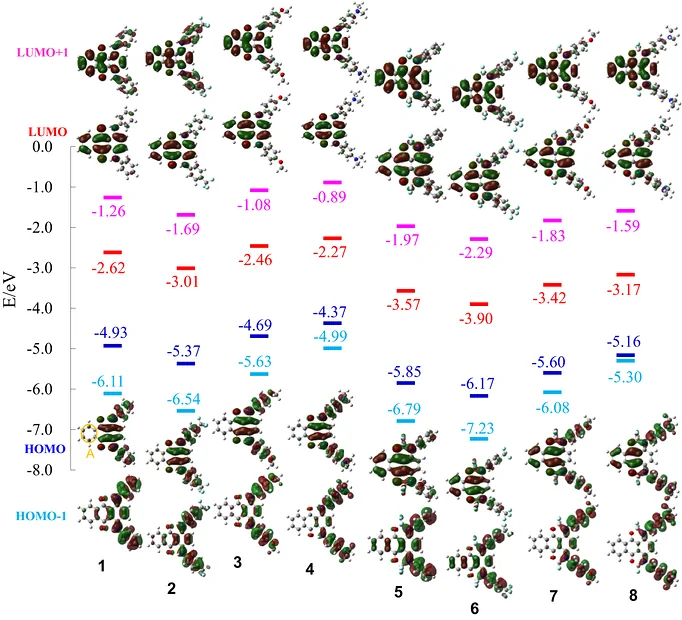
Molecular orbital energy diagrams and isodensity surface plots of **1**–**8**.

To obtain a deeper insight into the absorption properties, density functional theory (DFT) calculations were performed [65]. Geometry optimization was performed at the B3LYP/6‐31G(d,p) level. Electronic transitions were also calculated using the time dependent DFT (TDDFT) method [66, 67, 68] at the B3LYP/6‐31G(d,p) level. The molecular orbital energy diagrams and isodensity surface plots of **1**–**8** are shown in Figure 4. The calculated *λ*_max_ values, principal orbital transitions and oscillator strength (*f*) values of **1**–**8** are presented in Table 2. TDDFT calculations revealed that the S_0_−S_1_ transitions of compounds **1**–**8** are primarily attributed to HOMO–LUMO excitations. The LUMO orbitals of all compounds exhibit similar characteristics, being predominantly delocalized over the anthraquinone moiety. In contrast, the HOMO orbitals display significant differences between the 1,4‐bis(arylamino)anthraquinones **1**–**4** and their corresponding boron complexes **5**–**8**. For compounds **1**–**4**, the HOMO orbitals are delocalized over the aryl groups attached to the nitrogen atoms and only partially over the anthraquinone core. In contrast, the HOMO orbitals of the boron complexes **5**–**8** are delocalized over both the aryl groups on the nitrogen atoms and nearly the entire anthraquinone moiety. Consequently, unlike **5**–**8**, the HOMO orbitals of **1**–**4** exhibit negligible delocalization over the A moiety, as exemplified by compound **1** in Figure 4.

**TABLE 2:** Absorption maxima (*λ*_max_), oscillator strengths (*f*), and main orbital transition calculated by TDDFT at the B3LYP/6‐31G(d,p) level.

Compd	Transition	*λ*_max_ (nm)	Main orbital transition	*f*
**1**	S_0_ to S_1_	596	HOMO to LUMO (0.71)	0.23
	S_0_ to S_2_	424	HOMO‐1 to LUMO (0.68)	0.08
**2**	S_0_ to S_1_	588	HOMO to LUMO (0.71)	0.23
	S_0_ to S_2_	419	HOMO‐1 to LUMO (0.68)	0.07
**3**	S_0_ to S_1_	615	HOMO to LUMO (0.71)	0.24
	S_0_ to S_2_	464	HOMO‐1 to LUMO (0.70)	0.11
**4**	S_0_ to S_1_	655	HOMO to LUMO (0.71)	0.24
	S_0_ to S_2_	541	HOMO‐1 to LUMO (0.70)	0.13
**5**	S_0_ to S_1_	595	HOMO to LUMO (0.71)	0.26
	S_0_ to S_2_	448	HOMO‐1 to LUMO (0.70)	0.23
**6**	S_0_ to S_1_	594	HOMO to LUMO (0.71)	0.27
	S_0_ to S_2_	432	HOMO‐1 to LUMO (0.69)	0.25
**7**	S_0_ to S_1_	632	HOMO to LUMO (0.71)	0.27
	S_0_ to S_2_	550	HOMO‐1 to LUMO (0.70)	0.26
**8**	S_0_ to S_1_	704	HOMO to LUMO (0.71)	0.28
	S_0_ to S_2_	682	HOMO‐1 to LUMO (0.71)	0.32

The previously reported unsubstituted 1,5‐anthraquinone (*λ*_max_ = 530 nm, fwhm = 3,880 cm^−1^) exhibited a pronounced bathochromic shift of 164 nm in *λ*_max_ and a narrowing of the spectral features upon boron complexation (diboron complex: *λ*_max_ = 694 nm, fwhm = 2,510 cm^−1^) (Figure S3) [60]. In contrast, the corresponding unsubstituted 1,4‐anthraquinone **1** (*λ*_max_ = 640 nm, fwhm = 3,140 cm^−1^) also showed spectral narrowing upon boron complexation, but the *λ*_max_ underwent a hypsochromic shift (**5**: *λ*_max_ = 628 nm, fwhm = 2,280 cm^−1^) (Table 1). The observed spectral narrowing upon boron complexation is attributed to the rigidification of the anthraquinone skeleton. Both the S_0_–S_1_ (HOMO–LUMO) transitions of **1** and **5** correspond to intramolecular charge‐transfer (ICT) transitions from the two terminal aminophenyl groups to the anthraquinone moiety. The absence of a pronounced bathochromic shift upon boron complexation of **1** is attributed to the differences between the HOMOs of **1** and **5**. The lack of delocalization in the A portion of **1** suggests that its HOMO is more localized (Figure 4), resulting in a greater contribution of the ICT transition in **1** than in **5**. As a result, although boron complexation increases the electron‐withdrawing character of the anthraquinone moiety, the ICT contribution is reduced, leading to a slight hypsochromic shift of *λ*_max_.

Similarly, the trifluoromethyl derivative **2** (*λ*_max_ = 625 nm, fwhm = 3,310 cm^−1^) and the methoxy derivative **3** (*λ*_max_ = 639 nm, fwhm = 3,170 cm^−1^) exhibited spectral narrowing upon boron complexation, while their *λ*_max_ values remained nearly unchanged (**6**: *λ*_max_ = 628 nm, fwhm = 2,180 cm^−1^, **7**: *λ*_max_ = 637 nm, fwhm = 2,660 cm^−1^). On the other hand, the boron complexation of the dimethylamino derivative **4** (*λ*_max_ = 651 nm, fwhm = 3,440 cm^−1^) induced a bathochromic shift of *λ*_max_ along with spectral broadening (**8**: *λ*_max_ = 680 nm, fwhm = 4,900 cm^−1^). According to TDDFT calculations, the S_0_–S_1_ and S_0_–S_2_ transitions of **8** are located at similar wavelengths, and their overlap is considered to contribute to the observed spectral broadening (Table 2). The S_0_–S_2_ transitions of **5**, **6**, and **7** were observed at 414, 412, and 493 nm, respectively, whereas the S_0_–S_2_ transition of **8** was observed to overlap with the S_0_–S_1_ transition (Table 1). This suggests that the S_0_–S_2_ transition shifts to longer wavelengths as the electron‐donating ability of the substituent R increases. The S_0_–S_2_ transition of **5**–**8** was assigned to the HOMO–1 to LUMO excitation. Focusing on the HOMO−1 orbitals of **5**–**8**, the electron density in **5** and **6** is delocalized over the two aryl groups and the anthraquinone moiety, whereas in **7** and **8** bearing electron‐donating substituents, it is predominantly localized on the two aryl groups. In particular, this localization is most pronounced for **8** containing two dimethylamino groups. In contrast, no significant differences are observed in the LUMO orbitals of **5**–**8**. These calculation results suggest that, as the electron‐donating ability of substituent R increases, the contribution of ICT character to the S_0_–S_2_ (HOMO–1 to LUMO) transition becomes larger, leading to a red shift of the S_0_–S_2_ transition.

The cyclic voltammograms of **5**–**8** were measured in acetonitrile (Figure S4). For **5**, the first reduction half‐wave potential (*E*_1/2_ (red)) was −0.61 V (vs. Fc/Fc^+^). In acetonitrile, the conversion of *E*_1/2_ (vs. Fc/Fc^+^) to *E*_1/2_ (vs. NHE) is known to be as follows: *E*_1/2_ (vs. NHE) = *E*_1/2_ (vs. Fc/Fc^+^) + 0.63 V [69]. Therefore, the first *E*_1/2_ (red) value related to the LUMO energy level (*E*_LUMO_) of **5** was estimated to be 0.02 V (vs. NHE) (Table S1). The HOMO energy level (*E*_HOMO_) of **5** was estimated to be 1.87 V (vs. NHE) by adding the zero–zero excitation energy (E_0_‐_0_), estimated from the absorption onset wavelength in dichloromethane (670 nm, 1.85 eV), to the corresponding *E*_LUMO_ value (Table S1). Similarly, the *E*_HOMO_ and *E*_LUMO_ values for compounds **6**–**8** were estimated (Table S1). These values showed good correlation with those obtained from DFT calculations (Figure 4).

## 5 Fluorescence Properties

**FIGURE 5:**
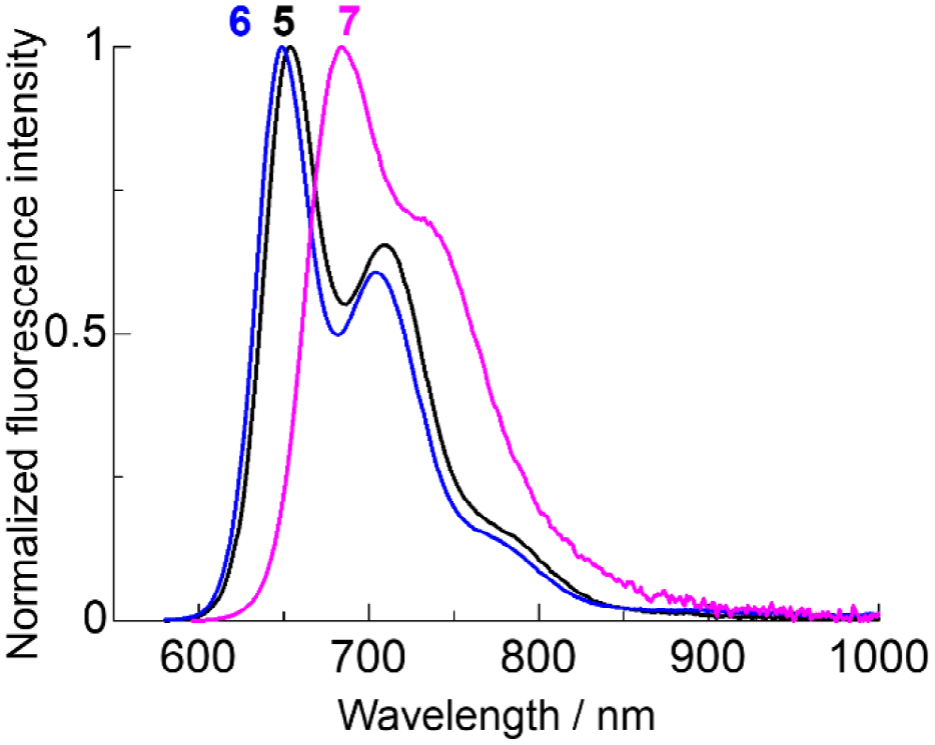
Normalized fluorescence spectra of 1,4‐diboron complexes in dichloromethane (3.0 × 10^−5^ M).

1,4‐Bis(arylamino)anthraquinones **1**–**4** exhibited no fluorescence. In contrast, among the boron complexes, the non‐, trifluoromethyl‐ and methoxy‐substituted derivatives **5**, **6**, and **7** showed fluorescence, whereas the dimethylamino‐substituted derivative **8** did not show fluorescence in dichloromethane (Figure 5). The maximum fluorescence wavelength (*F*_max_) **5**, **6**, and **7** were observed at 652, 648, and 683 nm, respectively (Table 3). The fluorescence spectra of **5** and **6** were mirror images of the absorption spectra with very small Stokes shifts, indicating only minor geometrical differences between the ground and excited states [70] (Figure S5). The observed peaks are attributable to vibrational levels, as in the absorption spectra. For example, in the fluorescence spectrum of **5**, the peaks at 650, 707, and 767 nm are assigned to the S_1,0_−S_0,0_, S_1,0_−S_0,1_, and S_1,0_−S_0,2_ transitions, respectively. The fluorescence quantum yield (*Φ*_f_) decreased as the electron‐donating ability of substituent R increased (**5**: *Φ*_f_ = 0.15, **6**: *Φ*_f_ = 0.25, **7**: *Φ*_f_ = 0.02, **8**: *Φ*_f_ = 0.00). Time‐resolved fluorescence decay measurements revealed fluorescence lifetimes (*τ*_s_) of **5**, **6** and **7** of 3.48, 5.38 and 0.46 ns, respectively, with their decay profiles well‐fitted by monoexponential functions. The radiative (*k*_f_) and nonradiative (*k*_nr_) rate constants were also calculated (Table 3). For compounds **5**–**7**, the *k*_f_ values were nearly identical (**5**: *k*_f_ = 0.043×10^9^ s^−1^, **6**: *k*_f_ = 0.046×10^9^ s^−1^, **7**: *k*_f_ = 0.043×10^9^ s^−1^), whereas the *k*_nr_ values were observed to decrease markedly as the electron‐donating ability of substituent R increased (**5**: *k*_nr_ = 0.24×10^9^ s^−1^, **6**: *k*_nr_ = 0.14×10^9^ s^−1^, **7**: *k*_nr_ = 2.1×10^9^ s^−1^). These results suggest that the decrease in the *Φ*_f_ value originates from the enhancement of nonradiative processes.

**TABLE 3:** Fluorescence properties of 1,4‐diboron complexes in dichloromethane^a^.

Compd	*F*_max_ (nm)	*Φ*_f_ ^b^, ^c^	*τ*_s_ ^d^ (ns)	*k*_f_ ^e^ (10^9^ s^−1^)	*k*_nr_ ^f^ (10^9^ s^−1^)
**5**	652, 706, 777	0.15	3.48	0.043	0.24
**6**	648, 701, 771	0.25	5.38	0.046	0.14
**7**	683, 740	0.02	0.46	0.043	2.1
**8**	—^g^				

^a^ Measured at a concentration of 3.0 × 10^−5^ M.

^b^ The excitation wavelengths (*λ*_ex_) were as follows: **5** (550 nm), **6** (550 nm) and **7** (550 nm).

^c^ Measured using an integrating sphere method.

^d^ Measured using a single‐photon‐counting method.

^e^ Radiative rate constant (*k*_f_ = *Φ*_f_/*τ*_s_).

^f^ Non‐radiative rate constant (*k*_nr_ = (1 – *Φ*_f_)/*τ*_s_).

^g^ Not observed.

ICT‐type dyes are generally known to exhibit decreased fluorescence intensity in polar solvents [71, 72]. To investigate the effect of solvent polarity, the fluorescence spectra of boron complexes **5**–**8** were measured in 1,4‐dioxane (relative permittivity = 2.2), which is less polar than dichloromethane (relative permittivity = 8.9). As a result, the dimethylamino‐substituted derivative **8** remained non‐emissive in 1,4‐dioxane, whereas **5**–**7** exhibited fluorescence (Figure S6). The *F*_max_ and *Φ*_f_ values were as follows (**5**: *F*_max_ = 654 nm, *Φ*_f_ = 0.16; **6**: *F*_max_ = 649 nm, *Φ*_f_ = 0.26; **7**: *F*_max_ = 684 nm, *Φ*_f_ = 0.03). Thus, trends similar to those observed in dichloromethane were observed. These results suggest that the non‐emissive nature of **8** and the relatively low *Φ*_f_ of **7** cannot be sufficiently explained solely by solvent polarity.

Next, to examine the effect of substituent rotation and vibration on fluorescence quenching, the fluorescence spectra of **5** and **7** were measured in mixed solvents of methanol (0.55 cP) and ethylene glycol (23.5 cP) with varying viscosity. For **5**, no significant change in *F*_max_ (645 nm) was observed with increasing ethylene glycol content, whereas the fluorescence intensity decreased (Figure S7a). This result suggests that changes in the solvent environment (including increased dielectric constant and specific interactions), rather than suppression of intramolecular rotation and vibration due to increased viscosity, may contribute to the observed quenching. For **7**, increasing the proportion of ethylene glycol resulted in a slight red shift of *F*_max_ from 674 to 680 nm, accompanied by a substantial decrease in fluorescence intensity (Figure S7b). This behavior is consistent with a greater contribution of the ICT transition in **7** compared to **5**, leading to enhanced sensitivity to changes in the solvent environment. These results suggest that intramolecular rotation and vibration do not play a major role in the relatively low fluorescence quantum yield observed upon introduction of the methoxy group.

**FIGURE 6:**
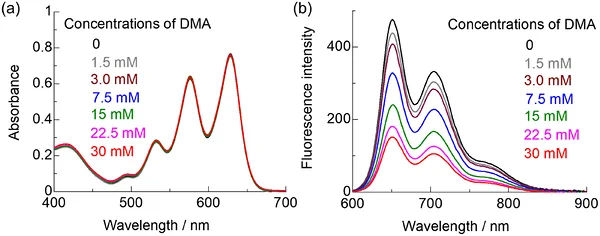
(a) UV−vis−NIR absorption and (b) fluorescence spectra of **5** in dichloromethane (3.0 × 10^−5^ M) with varying concentrations of DMA (0, 1.5 3.0 7.5, 15, 22.5 and 30 mM). Fluorescence spectra were recorded with an excitation wavelength of 500 nm.

**FIGURE 7:**
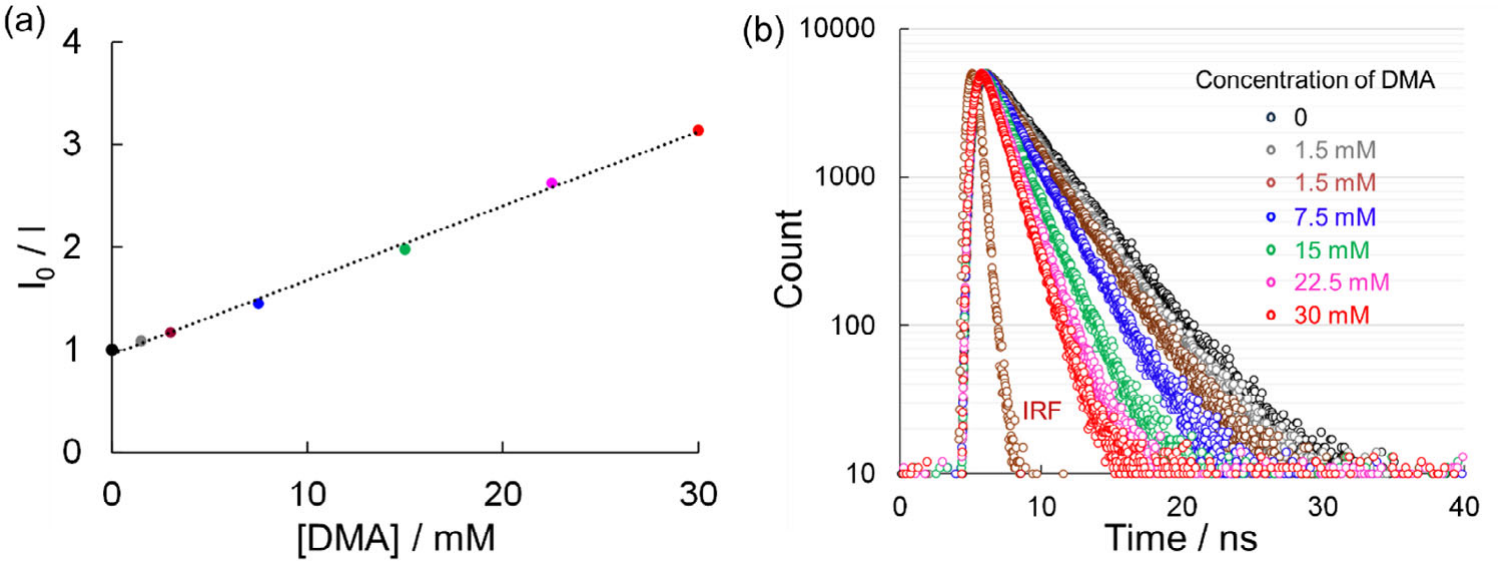
(a) Stern−Volmer plot for steady‐state fluorescence quenching of **5** by DMA. (b) Changes in the fluorescence decays of the **5** as a function of DMA concentration. Time‐resolved fluorescence measurements were performed at an excitation wavelength of 470 nm. The fluorescence decay curves were analyzed by reconvolution fitting using the measured instrument response function (IRF).

To examine the origin of the increase in the *k*_nr_ value with increasing electron‐donating ability of the substituent R, dimethylaminobenzene (DMA), an electron donor, was added to **5** in dichloromethane. No significant change was observed in the absorption spectra (Figure 6a). In contrast, the fluorescence intensity decreased with increasing amounts of DMA (Figure 6b). To clarify the fluorescence quenching mechanism, Stern–Volmer analyses were performed (Figure 7a). The Stern–Volmer plot exhibited good linearity (*R* ^2^ = 0.997), indicating the presence of a single dominant quenching process. From the slope of the Stern–Volmer plot, a Stern–Volmer quenching constant *K*_SV_ of 72.3 M^−1^ was obtained. Because no ground‐state interaction between DMA and **5** was detected in the absorption spectra (Figure 6a), static quenching is unlikely. Time‐resolved fluorescence decay measurements were then carried out to probe the excited‐state interaction (Figure 7b). The fluorescence lifetime of **5** was found to decrease progressively with increasing DMA concentration, demonstrating a dynamic quenching process. The measured *τ*_s_ values were 3.48, 3.19, 2.97, 2.46, 1.86, 1.49, and 1.27 ns at DMA concentrations of 0, 1.5, 3.0, 7.5, 15, 22.5, and 30 mM, respectively. On the basis of these results, the observed fluorescence quenching is attributed to dynamic intermolecular photoinduced electron transfer (PeT) from DMA to **5** [73, 74, 75]. This DMA quenching experiment serves as a model system for intermolecular electron transfer and supports the interpretation that the decrease in *Φ*_f_ observed for the 1,4‐diboron complexes with increasing electron‐donating ability of the substituent R originates from intramolecular PeT.

To obtain qualitative insight into the relative electron‐donating abilities of the donor moieties, the HOMO energies of the isolated donor units were calculated at the B3LYP/6‐31G(d,p) level. As shown in Figure S8, the calculated HOMO energies follow the order **8**> **7**> **5**> **6**, which correlates well with the experimentally observed fluorescence quenching efficiencies. These results support that the enhanced electron‐donating ability of the donor moiety facilitates intramolecular PeT upon photoexcitation.

**FIGURE 8:**
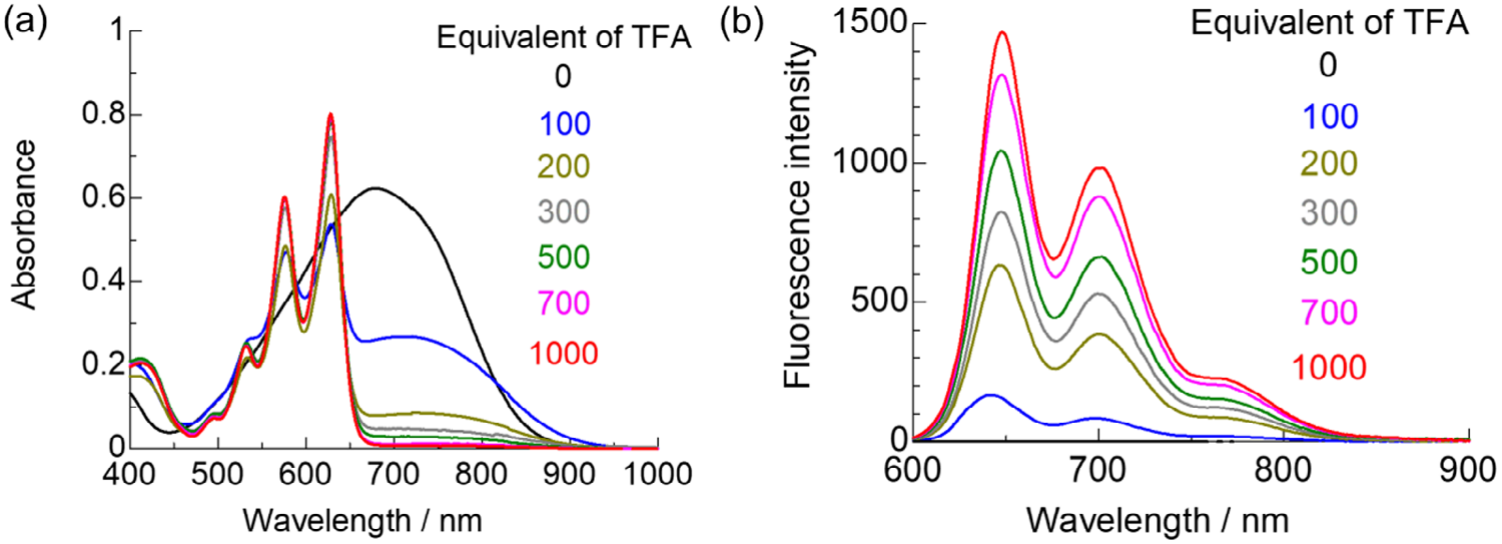
(a) UV−vis−NIR absorption and (b) fluorescence spectra of **8** in dichloromethane (3.0×10^−5^ M) with varying equivalents of TFA (0, 100, 200, 300, 500, 1000 equiv.).

Trifluoroacetic acid (TFA) was added to the nonfluorescent dimethylamino derivative **8** to investigate protonation of the dimethylamino groups. The UV−vis−NIR absorption spectra of **8** in dichloromethane upon addition of TFA are shown in Figure 8a. As the equivalents of added TFA increased, the absorption at 680 nm, assigned to **8**, decreased, and four new peaks appeared at 626, 575, 531, and 494 nm. The TDDFT calculations indicate that the S_0_−S_1_ transitions of the monoprotonated form **9’** (*λ*_max_ = 881 nm) and the diprotonated form **9** (*λ*_max_ = 603 nm) are both dominated by HOMO–LUMO transitions, exhibiting significant bathochromic and hypsochromic shifts, respectively, relative to **8** (*λ*_max_ = 704 nm) (Figure S9). On this basis, the newly emerged absorption bands at 626, 575, 531, and 494 nm upon addition of TFA are assigned to the S_0,0_−S_1,0_, S_0,0_−S_1,1_, S_0,0_−S_1,2_, and S_0,0_−S_1,3_ transitions of the diprotonated form **9**, respectively. The absence of an isosbestic point in the absorption spectra upon addition of TFA indicates the involvement of an intermediate species, namely, the monoprotonated form **9’** [76, 77]. Additionally, no absorption attributable to **9’** was observed in the longer‐wavelength region, suggesting that **9’** is rapidly converted to **9** upon formation. Furthermore, fluorescence at 648 nm emerged upon addition of TFA, with the intensity increasing as the amount of TFA was increased (Figure 8b). The excitation spectrum of **8** upon addition of excess TFA showed good agreement with the absorption spectrum of the diprotonated form **9** (Figure S10a). The fluorescence decay was well fitted with a single‐exponential function (Figure S10b). These results indicate that the observed emission predominantly originates from the diprotonated form **9**, with negligible contribution from **9’**. This fluorescence behavior is attributed to protonation of the dimethylamino groups, which converts the electron‐donating groups into electron‐withdrawing groups, thereby suppressing the intramolecular PeT process.

In our previous study, the 1,5‐diboron complex showed no fluorescence, even in the phenyl‐substituted derivative (Figure S3) [60]. Its *λ*_max_ value (694 nm) is shifted to longer wavelengths compared with the corresponding 1,4‐diboron complex **5** (628 nm). According to the energy gap law, the 1,5‐diboron complex is more prone to nonradiative deactivation than the 1,4‐diboron complex, which likely contributes to its nonfluorescent behavior [78, 79]. Additionally, DFT calculations suggest that the electronic structure of the 1,5‐diboron complex differs from that of the 1,4‐diboron complex, with the HOMO energy level being lower (Figure S11). These calculation results are considered to reflect differences in the electronic structures of the dye backbones. Taking into account the experimentally observed nonfluorescent behavior, in the 1,5‐diboron complex, not only the enhancement of nonradiative deactivation associated with the red shift of the absorption wavelength but also a nonradiative deactivation process related to intramolecular PeT may be involved [80, 81].

## 6 Cell Imaging

The potential of 1,4‐diboron complex **5** for cellular imaging was subsequently investigated. Due to its poor water solubility, we first measured the fluorescence spectrum in an aqueous medium containing a nonionic surfactant Cremophor EL (CrEL) [82, 83]. In this medium, **5** exhibited fluorescence with a maximum at 637 nm and vibronic peaks at 693 nm and approximately 760 nm (Figure S12), indicating that fluorescence was retained under these aqueous conditions.

**FIGURE 9:**
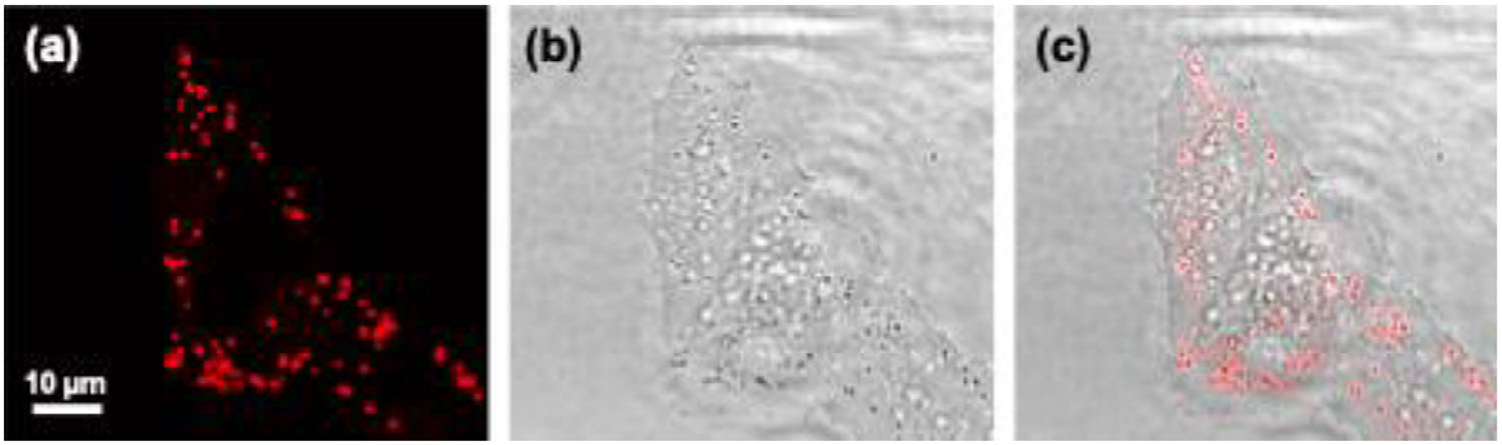
(a) Confocal fluorescence image of live HeLa cells stained with **5** (500 nM). Acquisition conditions: *λ*_ex_ = 561 nm and *λ*_em_ = 600–700 nm. (b) Bright field. (c) Merged image. Scale bar: 10 µm.

Following this, the staining behavior of **5** was evaluated in live HeLa cells. Cells were incubated with **5** (500 nM) in DMEM containing 10% fetal bovine serum (FBS) in a 5% CO_2_ incubator and then observed by confocal fluorescence microscopy without washing (Figure 9). Under these conditions, punctate intracellular fluorescence was readily observed even after 10 min incubation, demonstrating efficient cellular uptake of **5**. Co‐staining with LipiDye II showed substantial signal overlap, with a correlation coefficient of *R* = 0.89, indicating that, due to its hydrophobicity, **5** preferentially localizes to lipid droplets in living cells (Figure S13). In contrast, fluorescence images of fixed cells showed broadly distributed fluorescence, suggesting that fixation affects the subcellular distribution of **5** (Figure S14).

We also assessed the cytotoxicity of **5** using an MTT assay. After 24 h of incubation, a modest concentration‐dependent decrease in cell viability was observed at concentrations ranging from 0.25 to 2 µM (Figure S15). At the staining concentration used for imaging (500 nM), the reduction in viability remained limited, supporting the applicability of **5** for fluorescence imaging under the present conditions.

## 7 Conclusions

In summary, N^O‐type 1,4‐diboron complexes derived from 1,4‐bis(arylamino)anthraquinones exhibit near‐infrared fluorescence, in clear contrast to the previously reported non‐emissive 1,5‐diboron analogues. The fluorescence quantum yields decrease with increasing electron‐donating ability of the substituents, which is attributed to enhanced nonradiative decay via intramolecular PeT. This interpretation is supported by fluorescence quenching experiments using DMA and theoretical calculations, which reveal a correlation between electronic structure and nonradiative decay processes. In addition, protonation studies demonstrate that suppression of intramolecular PeT effectively restores fluorescence, further confirming its key role in the deactivation pathway. Comparison with 1,5‐diboron systems suggests that their non‐emissive nature originates from more efficient intramolecular PeT combined with energy gap law effects associated with red‐shifted absorption. These findings provide deeper insight into the structure–property relationship governing emissive behavior in diboron complexes of anthraquinone and offer useful guidelines for the molecular design of near‐infrared emissive materials.

## 8 Experimental Section

### 8.1 General Information

NMR spectra were recorded using JEOL ECX400P and ECA600 spectrometers. Infrared (IR) spectra were determined on a Simazu IR–Affinity spectrometer. UV–vis–NIR absorption spectra were recorded using a Hitachi U4100 and a Shimadzu UV‐3600i Plus spectrophotometer. Mass spectra were recorded on a JEOL JMS–700 spectrometer. Elemental analyses were performed on a Yanaco MT–6 CHN corder. Melting points were measured on a Yanagimoto MP–S2 micro–melting–point apparatus. Analytical thin‐layer chromatography (TLC) was performed on pre‐coated plates (Merck, silica gel 60 F254). Silica gel (Wakogel C–200) was used for column chromatography.

### 8.2 X‐Ray Crystallography

Single‐crystal X‐ray diffraction measurements were carried out with a Rigaku Mercury 375R/M CCD (XtaLAB‐mini) diffractometer with graphite‐monochromated Mo K*α* radiation (*λ* = 0.71075 Å). The crystal structure was solved using direct method with SHELXT‐2018/2 [84] and refined by full matrix least‐squaresmethods with anisotropic thermal parameters for nonhydrogen atoms on *F* ^2^ using SHELXL‐2018/3 [85]. Hydrogen atoms were placed at calculated positions and refined applying riding models. The Cambridge Crystallographic Data Center (CCDC) number for **5** is 2457602.

Crystallographic details for **5**: C_26_H_16_B_2_F_4_N_2_O_2_, *M* = 486.03 g mol^−1^; *T* = 293(2) K; monoclinic, space group P2_1_/n; *a* = 13.964(3), *b* = 9.580(2), *c* = 17.066(5) Å, *α* = 90, *β* = 101.196(10), *γ* = 90°; *V* = 2239.6(9) Å^3^; *Z* = 4; 2.974< *θ* < 27.594; *ρ*_calcd_ = 1.441 g cm^−3^; *μ* = 0.113 mm^−1^; *R*_int_ = 0.0611; *R*_1_ = 0.0595, w*R*_2_ = 0.1595 for 5003 reflections with *I* > 2*σ*(I) and 325 parameters; *S* = 1.027; CCDC 2457602.

### 8.3 Cell Experiments

#### 8.3.1 Cell Culture

HeLa (RCB0007) cells were obtained from RIKEN Cell Bank. The cells were cultured in Dulbecco's Modified Eagle's Medium (DMEM; low glucose, with *L*‐glutamine and sodium pyruvate; Wako, 041–29775) supplemented with 10% fetal bovine serum (FBS; Biosera, 554–02155) and 1% Antibiotic‐Antimycotic (AA; Wako, 161–23181) at 37 °C in a humidified 5% CO_2_ incubator. For imaging experiments, cells (2 × 10^4^ cells/dish) were seeded in glass‐bottom dishes or 96‐well glass plates two days before observation.

#### 8.3.2 Fluorescence Microscopy

*Live‐cell imaging*: Cells were incubated with **5** (500 nM) in DMEM(+) containing 1% DMSO for 10 min at 37 °C in a CO_2_ incubator and then subjected to confocal fluorescence imaging without washing. Fluorescence images were acquired with a FLUOVIEW FV3000 (Evident) confocal laser‐scanning microscope equipped with 405, 488, 561, 640, and 730 nm lasers, GaAsP PMT and GaAs PMT detectors, and a TruFocus Red Z‐drift compensator. For co‐staining experiments, cells were stained with **5** (500 nM) and 1 µM LipiDye II (Funakoshi). The acquisition parameters are given in the corresponding figure captions.

*Fixed‐cell imaging*: Cells were washed with PBS, fixed with 4% paraformaldehyde (PFA) in PBS at room temperature for 30 min, and washed three times with culture medium. The fixed cells were then stained with **5** (10 µM) in PBS containing Cremophor EL (CrEL) and imaged using a KEYENCE BZ‐X800 microscope.

### 8.4 MTT Assay

HeLa cells were seeded into 96‐well plates (2 × 10^3^ cells/well) and cultured in DMEM(+) at 37 °C for 2 days. The medium was then replaced with DMEM containing **5** at concentrations of 0, 0.25, 0.5, 1.0, and 2.0 µM (0.5% DMSO). After a 24 h incubation, MTT reagent was added to each well (final concentration: 0.5 mg/mL), and the plates were incubated for an additional 2 h. The wells were then washed with PBS to remove excess reagent, and the resulting formazan crystals were dissolved in DMSO (200 µL/well) with shaking for 10 min at room temperature. Absorbance at 530 nm was measured using a SpectraMax i3 plate reader (Molecular Devices). Results are expressed as percentages relative to the dye‐free control.

### 8.5 Synthesis of 1,4‐bis(Anilino)Anthraquinone (**1**) [61]

1,4‐dihydroxyanthraquinone (1.00 g, 4.16 mmol), aniline (4.26 g, 45.7 mmol), boric acid (100 mg, 1.62 mmol), zinc powder (60 mg, 0.92 mmol) and conc. HCl (0.40 mL, 12.9 mmol) were mixed and heated at 100°C for 16 h. The reaction mixture was quenched with water, extracted with DCM, dried over Na_2_SO_4_ and evaporated under reduced pressure. Column chromatography of the residue on silica gel (DCM: hexane = 1:1) gave **1** (550 mg, Y = 34%) as a purple solid. ^1^H NMR (400 MHz, CDCl_3_) *δ* 7.17 (t, *J* = 7.3 Hz, 2H), 7.28 (d, *J* = 7.3 Hz, 4H), 7.39 (dd, *J* = 7.3, 7.3 Hz, 4H), 7.51 (s, 2H), 7.77 (dd, *J* = 5.7, 3.7 Hz, 2H), 8.39 (dd, *J* = 5.7, 3.7 Hz, 2H), 12.2 (s, 2H); ^13^C NMR (100 Hz, CDCl_3_) *δ* 111.61, 123.82, 123.96, 124.65, 124.77, 124.87, 124.92, 126.29, 126.38, 129.47, 129.56, 132.72, 134.34, 139.53, 143.78, 183.52; IR (KBr) 1597, 1578, 1558, 1504, 1366, 1261, 1169, 1072, 1026, 729, 689 cm^−1^.

### 8.6 Synthesis of 1,4‐bis(4‐trifluoromethylanilino)Anthraquinone (**2**)

1,4‐dihydroxyanthraquinone (502 mg, 2.09 mmol), 4‐trifluoromethylaniline (3.72 g, 23.1 mmol), boric acid (51 mg, 0.83 mmol), zinc powder (30 mg, 0.46 mmol) and conc. HCl (0.40 mL, 12.9 mmol) were mixed and heated at 100°C for 5 h. The reaction mixture was quenched with water, extracted with DCM, dried over Na_2_SO_4_ and evaporated under reduced pressure. Column chromatography of the residue on silica gel (DCM: hexane = 1:1) gave **4** (180 mg, Y = 16%) as a purple solid. mp: 284‒285°C; ^1^H NMR (400 MHz, CDCl_3_) *δ* 7.36 (d, *J* = 8.0 Hz, 4H), 7.63 (d, *J* = 8.0 Hz, 4H), 7.63 (s, 2H), 7.79 (dd, *J* = 6.0, 3.3 Hz, 2H), 8.35 (dd, *J* = 6.0, 3.3 Hz, 2H), 12.1 (s, 2H); ^13^C NMR (100 MHz, CDCl_3_) *δ* 113.57, 121.99, 122.10, 124.13 (q, *J* = 270.8 Hz), 124.74, 125.74 (q, *J* = 32.4 Hz), 126.53, 126.62, 126.81, 133.40, 134.02, 142.15, 143.14, 184.55; ^19^F NMR (376 MHz, CDCl_3_) *δ* ‐61.91 (s, 6F); IR (KBr) 1612, 1578, 1512, 1323, 1265, 1161, 1111, 1072, 829, 729 cm^−1^; HRMS (FAB) *m*/*z* calcd for C_28_H_17_F_6_N_2_O_2_ [M + H]^+^ 527.1189, found 527.1151.

### 8.7 Synthesis of 1,4‐bis(4‐methoxyanilino)Anthraquinone (**3**) [62]

1,4‐dihydroxyanthraquinone (1.02 g, 4.25 mmol), 4‐methoxyaniline (5.65 g, 45.9 mmol), boric acid (101 mg, 1.63 mmol), zinc powder (62 mg, 0.95 mmol) and conc. HCl (0.40 mL, 12.9 mmol) were mixed and heated at 100°C for 5 h. The reaction mixture was quenched with water, extracted with DCM, dried over Na_2_SO_4_ and evaporated under reduced pressure. Column chromatography of the residue on silica gel (DCM) gave **3** (887 mg, Y = 46%) as a purple solid. ^1^H NMR (400 MHz, CDCl_3_) *δ* 3.83 (s, 6H), 6.93 (d, *J* = 8.9 Hz, 4H), 7.19 (d, *J* = 8.9 Hz, 4H), 7.31 (s, 2H), 7.76 (dd, *J* = 6.0, 3.2 Hz, 2H), 8.40 (dd, *J* = 6.0, 3.2 Hz, 2H), 12.2 (s, 2H); ^13^C NMR (100 Hz, CDCl_3_) *δ* 55.45, 55.58, 110.59, 114.68, 114.45, 124.88, 126.28, 126.39, 132.16, 132.45, 134.45, 144.93, 157.25, 182.95; IR (KBr) 1597, 1578, 1558, 1512, 1362, 1296, 1250, 1165, 1072, 1038, 818, 725 cm^−1^.

### 8.8 Synthesis of 1,4‐bis(4‐dimethylaminoanilino)Anthraquinone (**4**) [63]

1,4‐dihydroxyanthraquinone (2.00 g, 8.32 mmol), *N*,*N*‐dimethyl‐*p*‐phenylenediamine (8.52 g, 62.6 mmol), boric acid (200 mg, 3.24 mmol), zinc powder (120 mg, 1.84 mmol) and conc. HCl (0.80 mL, 25.8 mmol) were mixed and heated at 100°C for 10 h. The reaction mixture was quenched with water, extracted with DCM, dried over Na_2_SO_4_ and evaporated under reduced pressure. Column chromatography of the residue on silica gel (DCM: EtOAc = 10:1) gave **2** (1.67 g, Y = 42%) as a blue solid. ^1^H NMR (400 MHz, CDCl_3_) *δ* 2.97 (s, 12H), 6.75 (d, *J* = 8.8 Hz, 4H), 7.14 (d, *J* = 8.8 Hz, 4H), 7.31 (s, 2H), 7.74 (dd, *J* = 5.8, 3.8 Hz, 2H), 8.41 (dd, *J* = 5.8, 3.8 Hz, 2H), 12.3 (s, 2H); ^13^C NMR (100 Hz, CDCl_3_) *δ* 40.76, 40.85, 110.07, 113.22, 125.21, 126.20, 126.31, 128.36, 132.16, 134.57, 145.54, 148.54, 182.31; IR (KBr) 1613, 1585, 1547, 1520, 1504, 1366, 1261, 1165, 1134, 826, 729 cm^−1^.

### 8.9 Synthesis of **5**

Boron trifluoride diethyl ether complex (2 mL, 16 mmol) were added to a solution of **1** (390 mg, 1.00 mmol) in *o‐*xylene (300 mL). After refluxing for 1 day, the reaction mixture was quenched with water, extracted with DCM, dried over Na_2_SO_4_ and evaporated under reduced pressure. Column chromatography of the residue on silica gel (DCM) gave **5** (507 mg, Y = 80%) as a blue solid.

mp: 285‒286°C; ^1^H NMR (400 MHz, CDCl_3_) *δ* 7.08 (s, 2H), 7.36 (d, *J* = 6.9 Hz, 4H), 7.44−7.52 (m, 6H), 7.95 (dd, *J* = 7.9, 3.2 Hz, 2H), 8.72 (dd, *J* = 7.9, 3.2 Hz, 2H); ^13^C NMR (100 Hz, CDCl_3_) *δ* 125.17, 126.81, 129.13, 129.71, 130.46, 132.50, 133.17, 138.41, 152.68, 154.18, 159.40; ^11^B NMR (128 MHz, CDCl_3_) *δ* 0.35 (t, *J* = 13.5 Hz, 2B); ^19^F NMR (376 MHz, CDCl_3_) *δ* ‐127.8 (q, *J* = 13.5 Hz, 4F); IR (KBr) 1582, 1524, 1485, 1308, 1227, 1184, 1141, 1111, 1045, 1003, 698 cm^−1^; EIMS (70 eV) *m/z* (rel intensity) 448 (13), 486 (M^+^, 100); Anal. Found: C, 64.04; H, 3.55; N, 5.84%. Calcd for C_26_H_16_B_2_F_4_N_2_O_2_: C, 64.25; H, 3.32; N, 5.76%.

### 8.10 Synthesis of **6**

Boron trifluoride diethyl ether complex (2 mL, 16 mmol) were added to a solution of **2** (526 mg, 1.00 mmol) in *o‐*xylene (300 mL). After refluxing for 1 day, the reaction mixture was quenched with water, extracted with DCM, dried over Na_2_SO_4_ and evaporated under reduced pressure. Column chromatography of the residue on silica gel (DCM) gave **6** (423 mg, Y = 68%) as a blue solid.

mp: 298‒299°C; ^1^H NMR (400 MHz, CDCl_3_) *δ* 7.08 (s, 2H), 7.51 (d, *J* = 8.2 Hz, 4H), 7.79 (d, *J* = 8.2 Hz, 4H), 7.99 (dd, *J* = 6.4, 3.3 Hz, 2H), 8.74 (dd, *J* = 6.4, 3.3 Hz, 2H); ^13^C NMR (150 MHz, CDCl_3_) *δ* 123.46 (q, *J* = 270.1 Hz), 127.00, 127.03, 127.28, 127.56, 130.61, 131.51 (q, *J* = 33.1 Hz), 132.52, 133.91, 141.50, 149.40, 153.84, 161.01; ^11^B NMR (128 MHz, CDCl_3_) *δ* 0.33 (t, *J* = 14.7 Hz, 2B); ^19^F NMR (376 MHz, CDCl_3_) *δ* ‐126.83 (q, *J* = 14.7 Hz, 4F), ‐62.66 (s, 6F); IR (KBr) 1582, 1523, 1485, 1327, 1169, 1130, 1111, 1069, 1049 cm^−1^; EIMS (70 eV) *m/z* (rel intensity) 622 (M^+^, 18), 584 (100), 526 (51), 439 (30); Anal. Found: C, 54.18; H, 2.30; N, 4.63%. Calcd for C_28_H_14_B_2_F_10_N_2_O_2_: C, 54.06; H, 2.27; N, 4.50%.

### 8.11 Synthesis of **7**

Boron trifluoride diethyl ether complex (2 mL, 16 mmol) were added to a solution of **3** (450 mg, 1.00 mmol) in *o‐*xylene (300 mL). After refluxing for 5 h, the reaction mixture was quenched with water, extracted with DCM, dried over Na_2_SO_4_ and evaporated under reduced pressure. Column chromatography of the residue on silica gel (DCM) gave **7** (448 mg, Y = 82%) as a blue solid.

Melting point (mp) >300°C; ^1^H NMR (400 MHz, CDCl_3_) *δ* 3.86 (s, 6H), 7.01 (d, *J* = 9.0 Hz, 4H), 7.15 (s, 2H), 7.29 (d, *J* = 9.0 Hz, 4H), 7.93 (dd, *J* = 6.2, 3.5 Hz, 2H), 8.71 (dd, *J* = 6.2, 3.5 Hz, 2H); ^13^C NMR (100 Hz, CDCl_3_) *δ* 55.57, 55.71, 114.84, 114.96, 126.68, 126.75, 127.81, 127.93, 130.37, 131.22, 132.38, 132.94, 154.24, 158.80, 160.06; ^11^B NMR (128 MHz, CDCl_3_) *δ* 0.33 (t, *J* = 12.3 Hz, 2B); ^19^F NMR (376 MHz, CDCl_3_) *δ* ‐128.7 (q, *J* = 12.3 Hz, 4F); IR (KBr) 1605, 1582, 1508, 1477, 1300, 1254, 1180, 1138, 1107, 1053, 1026, 995, 841 cm^−1^; EIMS (70 eV) *m/z* (rel intensity) 368 (16), 508 (28), 546 (M^+^, 100); Anal. Found: C, 61.24; H, 3.73; N, 5.18%. Calcd for C_28_H_20_B_2_F_4_N_4_O_2_: C, 61.58; H, 3.69; N, 5.13%.

### 8.12 Synthesis of **8**

Boron trifluoride diethyl ether complex (2 mL, 16 mmol) were added to a solution of **4** (477 mg, 1.00 mmol) in *o‐*xylene (300 mL). After refluxing for 5 h, the reaction mixture was quenched with water, extracted with DCM, dried over Na_2_SO_4_ and evaporated under reduced pressure. Column chromatography of the residue on silica gel (DCM) gave **8** (475 mg, Y = 83%) as a blue solid.

Melting point (mp) >300°C; ^1^H NMR (400 MHz, CDCl_3_) *δ* 3.03 (s, 12H), 6.76 (d, *J* = 8.8 Hz, 4H), 7.22 (s, 2H), 7.25 (d, *J* = 8.8 Hz, 4H), 7.88 (dd, *J* = 6.3, 3.3 Hz, 2H), 8.70 (dd, *J* = 6.3, 3.3 Hz, 2H); ^13^C NMR (100 Hz, CDCl_3_) *δ* 40.34, 40.43, 112.30, 126.30, 126.36, 127.45, 127.56, 127.70, 130.11, 131.52, 132.19, 134.57, 150.61, 153.52, 157.08; ^11^B NMR (128 MHz, CDCl_3_) *δ* 0.31 (t, *J* = 13.5 Hz, 2B); ^19^F NMR (376 MHz, CDCl_3_) *δ* ‐131.2 (q, *J* = 13.5 Hz, 4F); IR (KBr) 1601, 1520, 1481, 1366, 1300, 1188, 1107, 1045 cm^−1^; EIMS (70 eV) *m/z* (rel intensity) 572 (M^+^, 61), 534 (100); Anal. Found: C, 62.66; H, 4.23; N, 9.76%. Calcd for C_30_H_26_B_2_F_4_N_4_O_2_: C, 62.97; H, 4.58; N, 9.79%.

## Conflicts of Interest

The authors declare no conflicts of interest.

## Supporting information




**Supporting file 1**: chem71179‐sup‐0001‐SuppMat.pdf

**Supporting file 2**: chem71179‐sup‐0002‐cif.zip

## Data Availability

The data that supports the findings of this study are available in the supplementary material of this article
