# Supplementary material for: Synthesis and Optical Properties of Diboron Complexes of 1,4‐Anthraquinone Derivatives
Source: Chemistry. 2026 May 25;32(34):e71179. doi: 10.1002/chem.71179 (PMC13569954; doi:10.1002/chem.71179)
Supplement: Supplementary file 1 [file CHEM-32-e71179-s002.pdf]

Supporting information

Synthesis and Optical Properties of Diboron Complexes of 1,4-Anthraquinone Derivatives

Yasuhiro Kubota,*[a] Ayumi Ogasawara,[a] Ayato Hakamada,[b] Naoya Suzuki,[c] Masayasu Taki,[c] Shota Mizuno,[a] Toshiyasu Inuzuka,[d] Kazumasa Funabiki,[a] Chie Yamada-Kubota,[e] Miho Kuwahara[e] and Tetsuya Hara[e]

[a]Department of Chemistry and Biomolecular Science, Faculty of Engineering, Gifu University, 1-1 Yanagido, Gifu, 501-1193, Japan

[b]Department of Chemistry, Graduate School of Science, Nagoya University, Furo, Chikusa, Nagoya 464-8602, Japan

[c]Institute for Glyco-core Research (iGCORE), Gifu University, 1-1 Yanagido, Gifu 501-1193, Japan

[d]Life Science Research Center, Gifu University, 1-1 Yanagido, Gifu 501-1193, Japan

[e]Department of Occlusal and Oral Functional Rehabilitation, Graduate School of Medicine, Dentistry, and Pharmaceutical Sciences, Okayama University, 2-5-1 Shikata-cho, Kita-ku, Okayama 700-8525, Japan

Contents

1. Experimentally obtained bond lengths of **5** (**Figure S1**)
2. Resonance structures between the *β*-iminoenolate and *β*-ketoiminate forms (**Figure S2**)
3. The previously reported 1,5-diboron complex bearing phenyl groups on the nitrogen atoms (**Figure S3**)
4. Cyclic voltammograms of **5**–**8** measured in acetonitrile (**Figure S4**)
5. Electrochemical properties of 1,4-diboron dyes (**Table S1**)
6. Mirror-image relationship between the absorption and fluorescence spectra of **5** (**Figure S5**)
7. Normalized fluorescence spectra of 1,4-diboron complexes in 1,4-dioxane (**Figure S6**)
8. Fluorescence spectra of **5** and **7** in MeOH–ethylene glycol mixtures with different volume fractions of ethylene glycol (**Figure S7**)

9. HOMO energies of the isolated donor moieties (**Figure S8**)

10. Protonation of dimethylamino-substituted 1,4-diboron complex **8** (**Figure S9**)

11. Excitation spectrum and fluorescence decay of **8** upon addition of 1000 equivalents of TFA (**Figure S10**)

12. Molecular orbital energy diagrams and isodensity surface plots of the isolated acceptor moieties. (**Figure S11**)

13. Normalized fluorescence spectra of **5** in aqueous formulated solution (**Figure S12**)

14. Confocal fluorescence images of live HeLa cells co-stained with **5** and LipiDye II (**Figure S13**)

15. Widefield fluorescence images of fixed HeLa cells incubated with **5** (**Figure S14**)

16. Cell viability of HeLa cells after 24 h incubation with **5** determined by the MTT assay (**Figure S15**)

17. NMR spectra of **1–8** (**Figure S16–S31**)

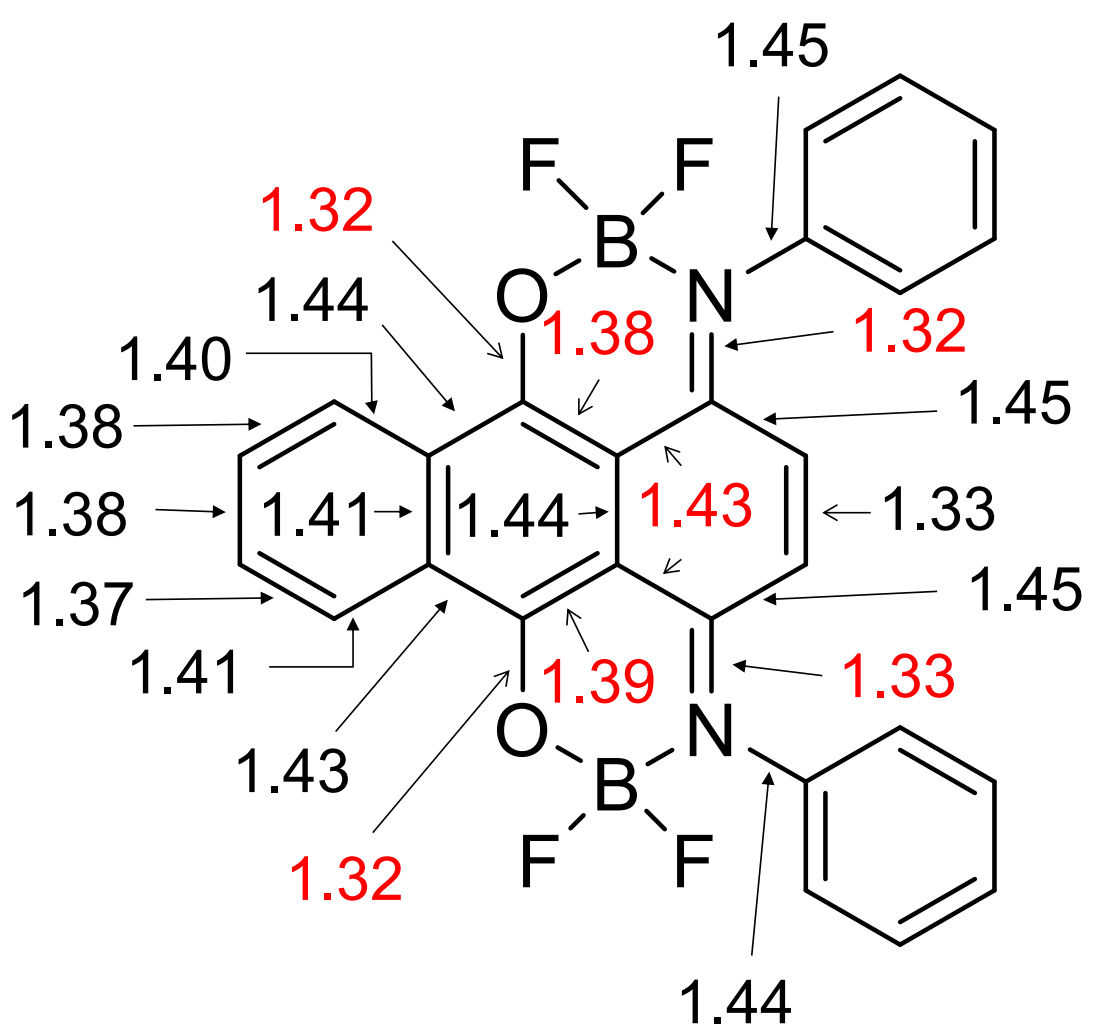

Figure S1. Experimentally obtained bond lengths of **5**. The unit of bond length is Å.

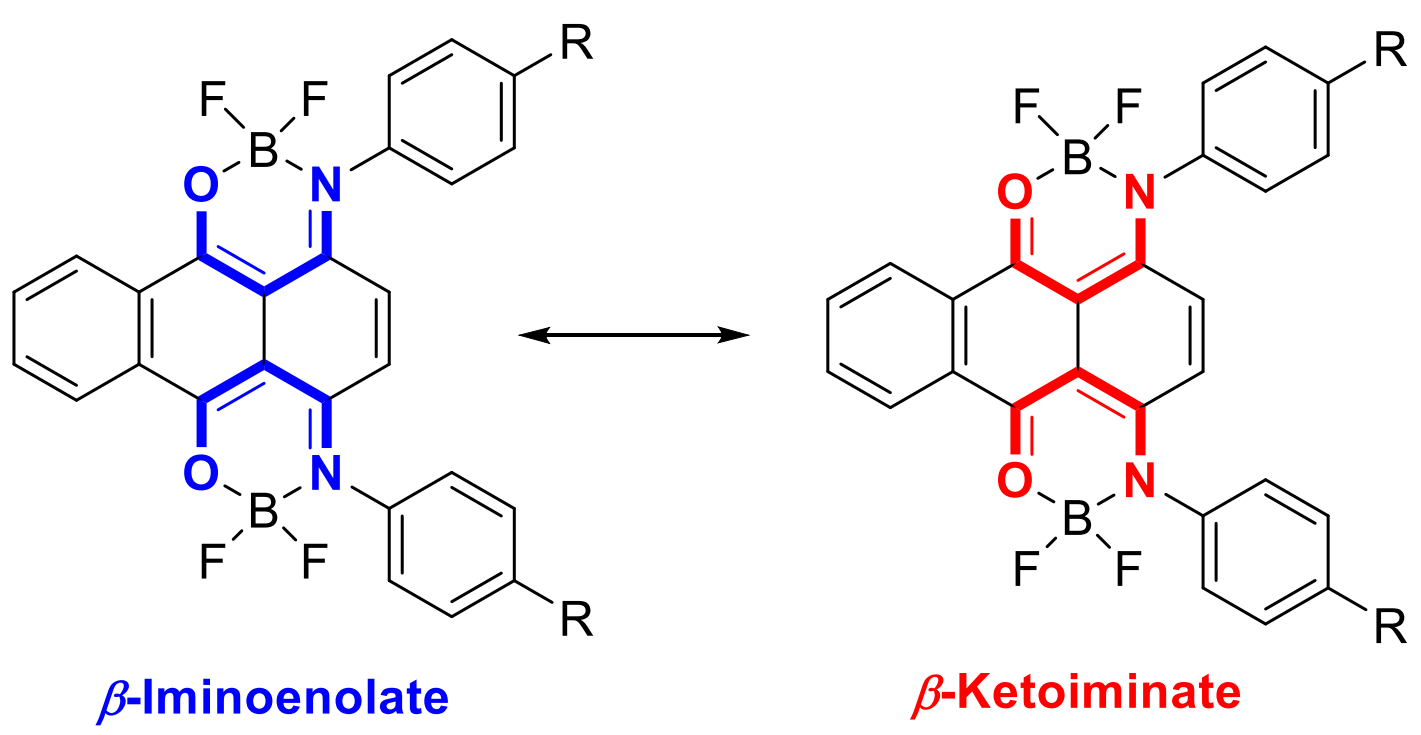

Figure S2. Resonance structures between the *β*-iminoenolate and *β*-ketoiminate forms.

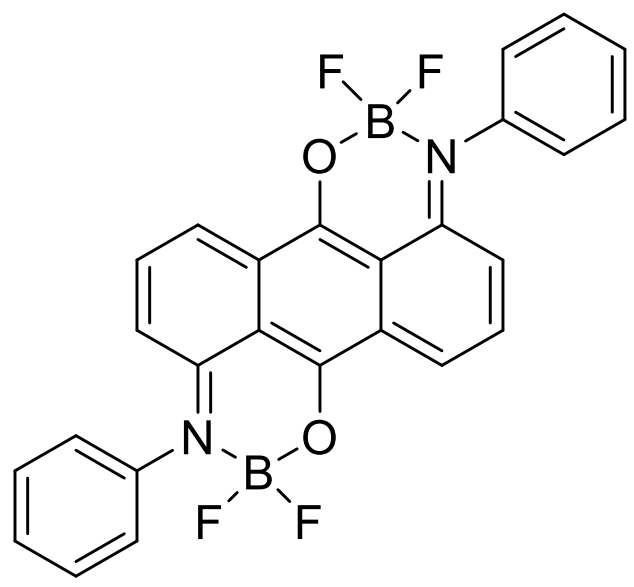

Figure S3. The previously reported 1,5-diboron complex bearing phenyl groups on the nitrogen atoms[26].

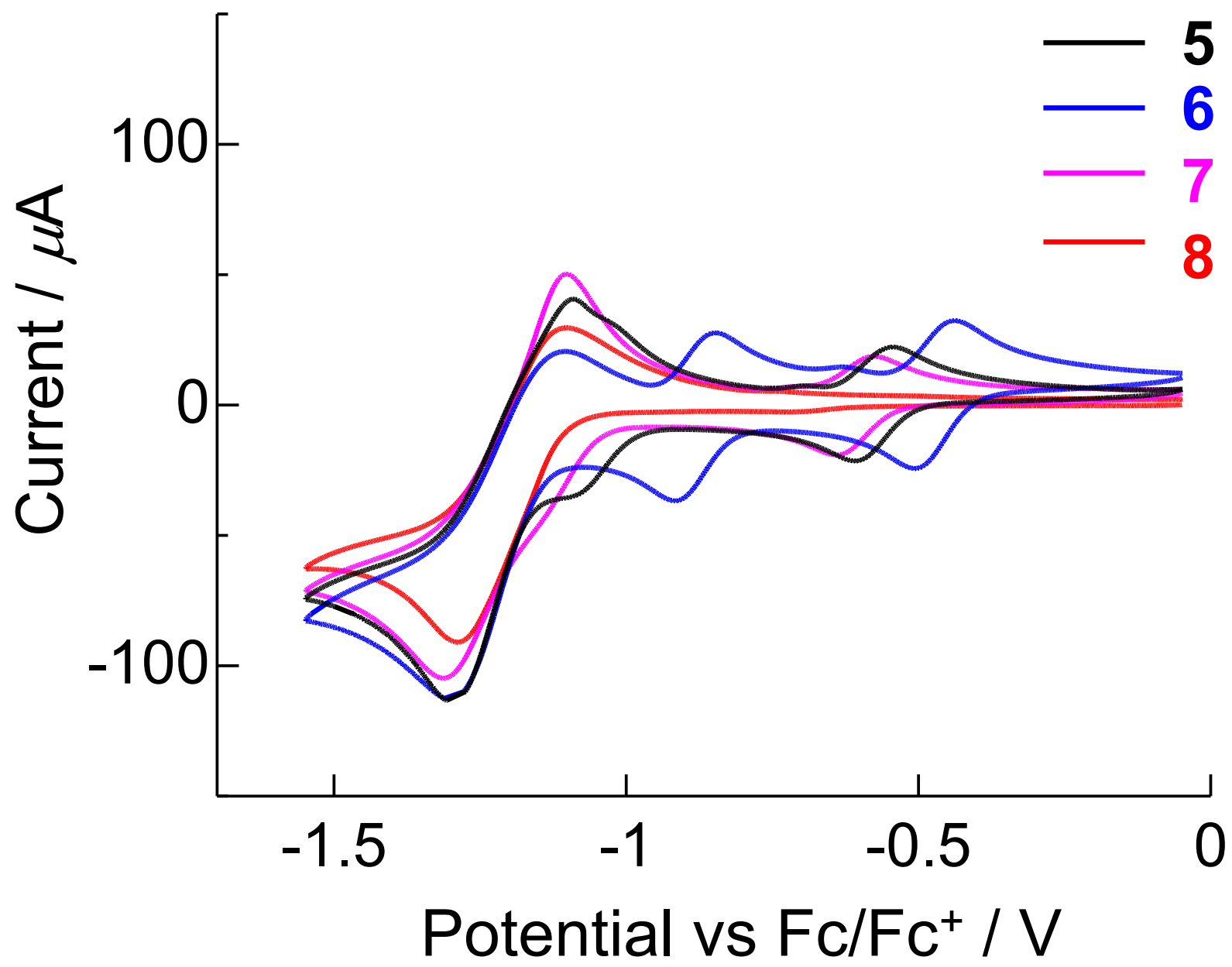

Figure S4. Cyclic voltammograms of **5–8** measured in acetonitrile containing tetrabutylammonium perchlorate (0.1 M) as a supporting electrolyte. AgQRE, a platinum wire, and a carbon electrode were employed as the reference, counter, and working electrodes, respectively. The scan rate was 100 mV s^{-1}. Fc/Fc^+ was used as an external reference.

Table S1. Electrochemical properties of 1,4-diboron dyes

Compd	$E_{HOMO}{}^a$ / V vs NHE	$E_{0\text{-}0}{}^b$ / eV	$E_{LUMO}{}^c$ / V vs NHE
**5**	1.87	1.85	0.02
**6**	1.99	1.86	0.13
**7**	1.76	1.77	-0.01
**8**	1.33	1.38	-0.05

[a]The first half-wave potentials of reduction were measured in acetonitrile with tetrabutylammonium perchlorate (0.1 M) with a scan rate of 100 mV s^{-1} vs Fc/Fc^+ and then converted to vs NHE by adding 0.63 V[33]. [b]The zero-zero excitation energies were estimated from the absorption onset wavelength in dichloromethane. [c]The HOMO energy levels were obtained by adding the $E_{0\text{-}0}$ values to the corresponding E_{LUMO} values.

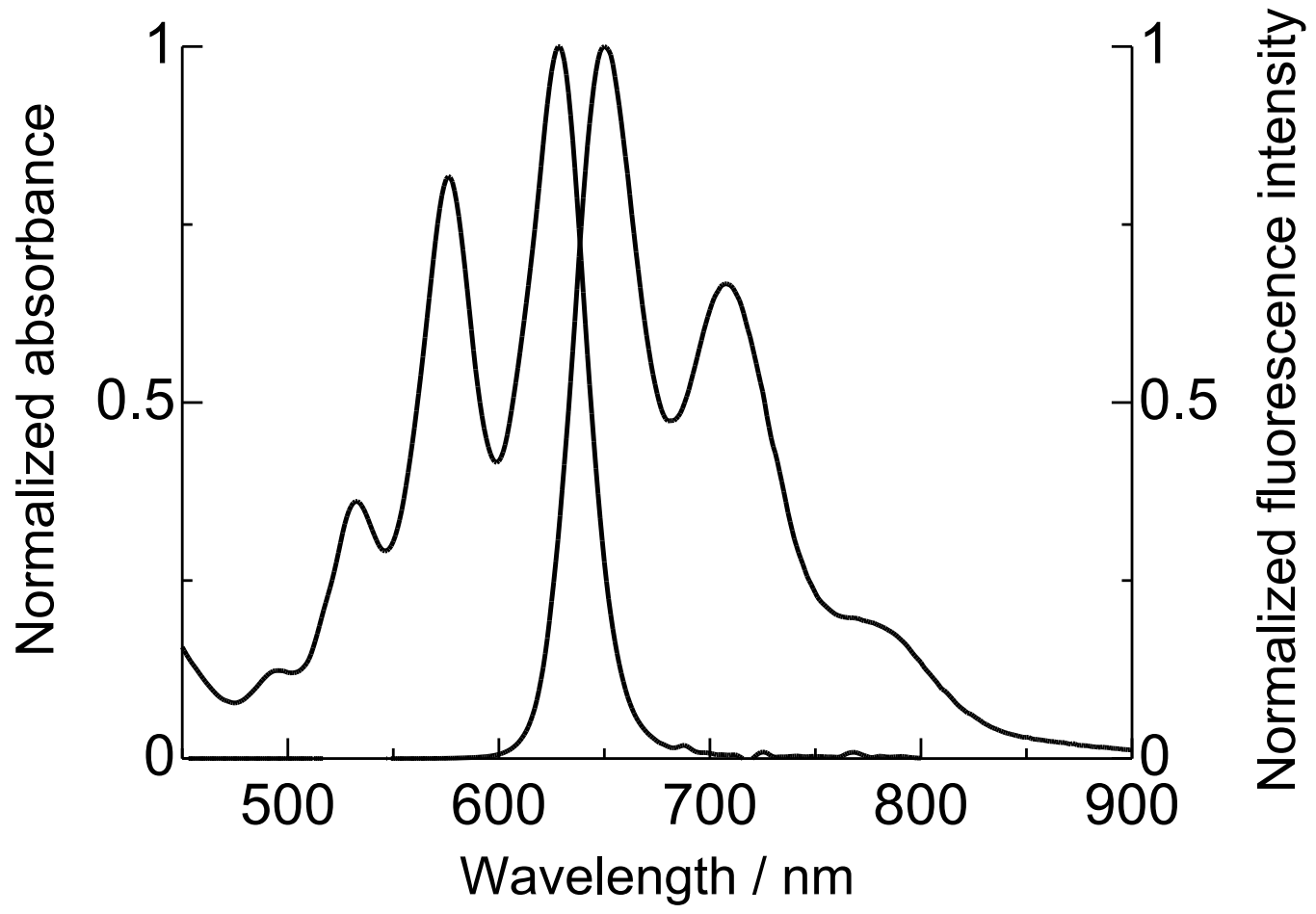

Figure S5. Mirror-image relationship between the absorption and fluorescence spectra of **5**.

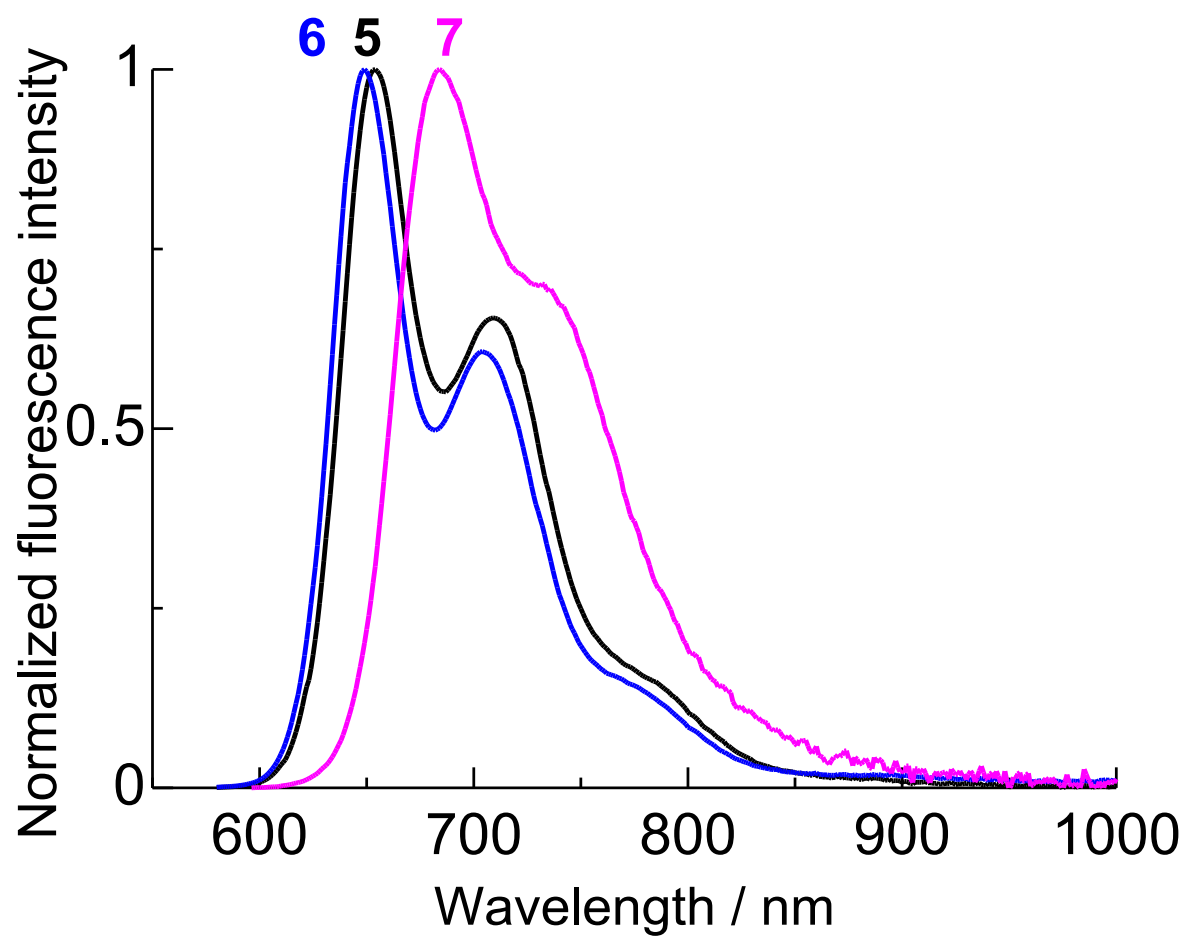

Figure S6. Normalized fluorescence spectra of 1,4-diboron complexes in 1,4-dioxane.

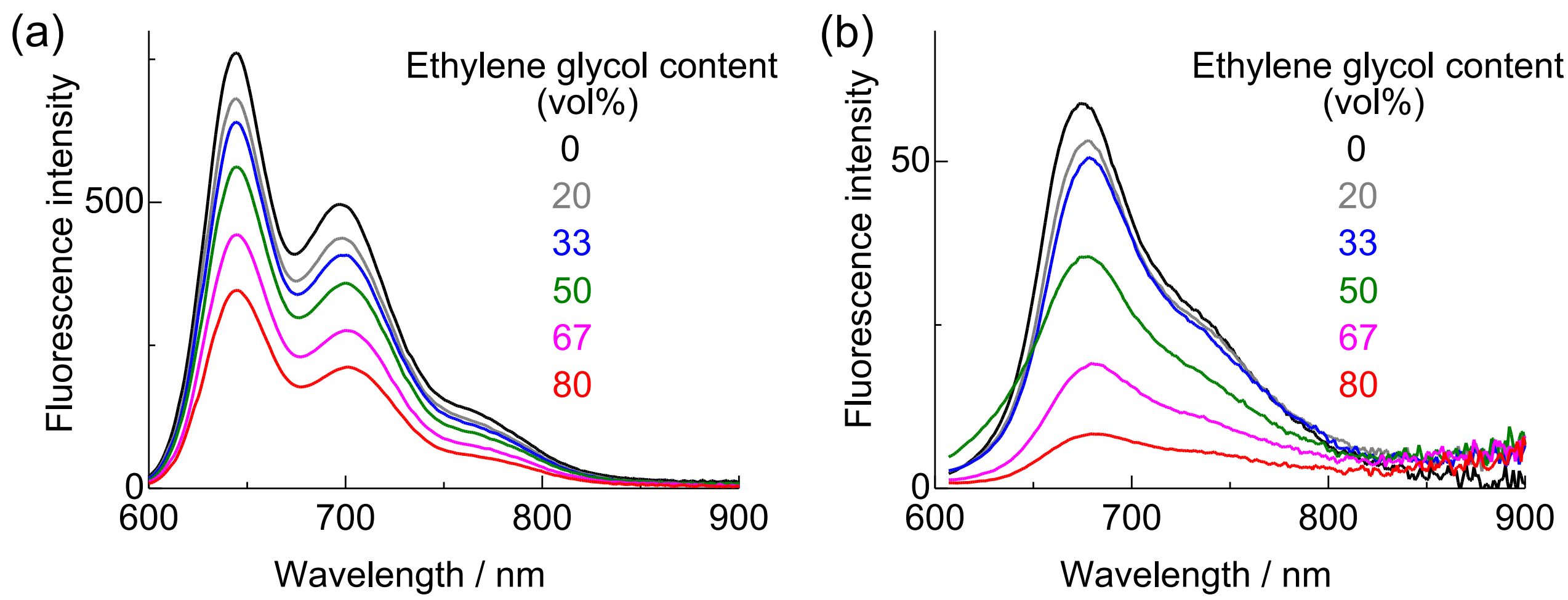

Figure S7. Fluorescence spectra of (a) **5** and (b) **7** in MeOH–ethylene glycol mixtures with different volume fractions of ethylene glycol (3.0×10^{-5} M).

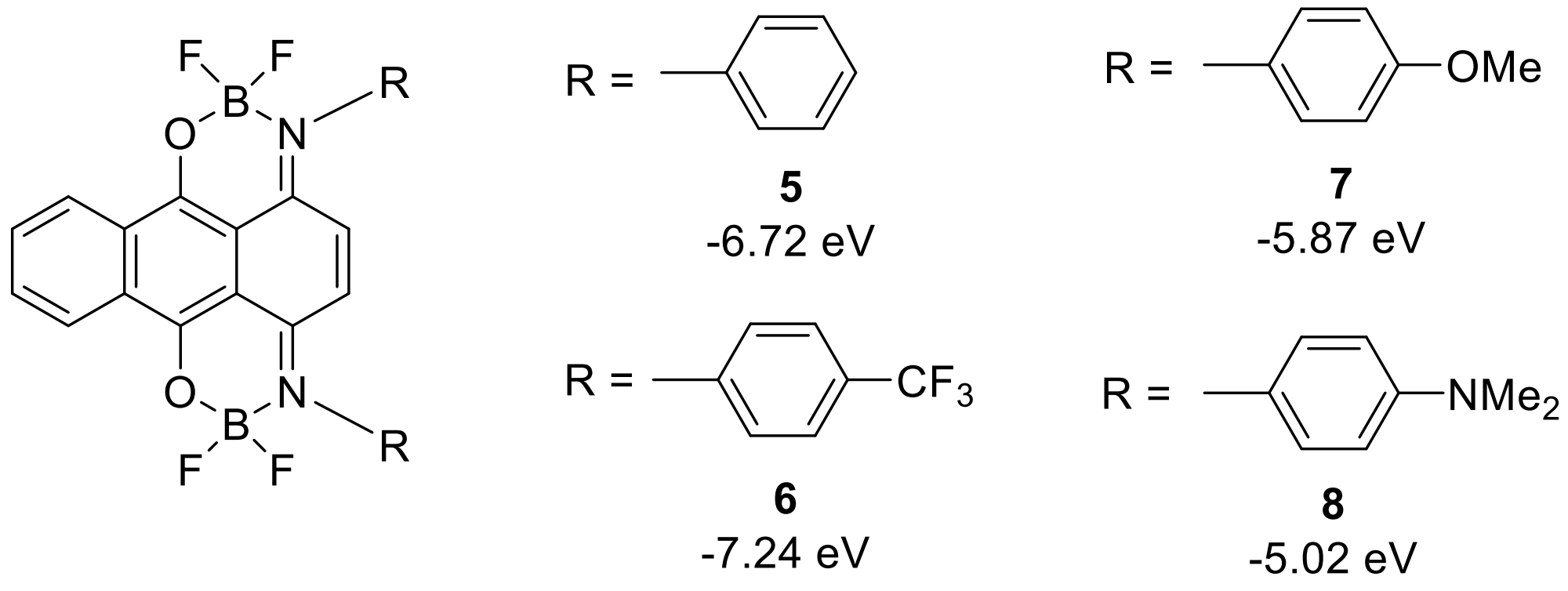

Figure S8. HOMO energies of the isolated donor moieties.

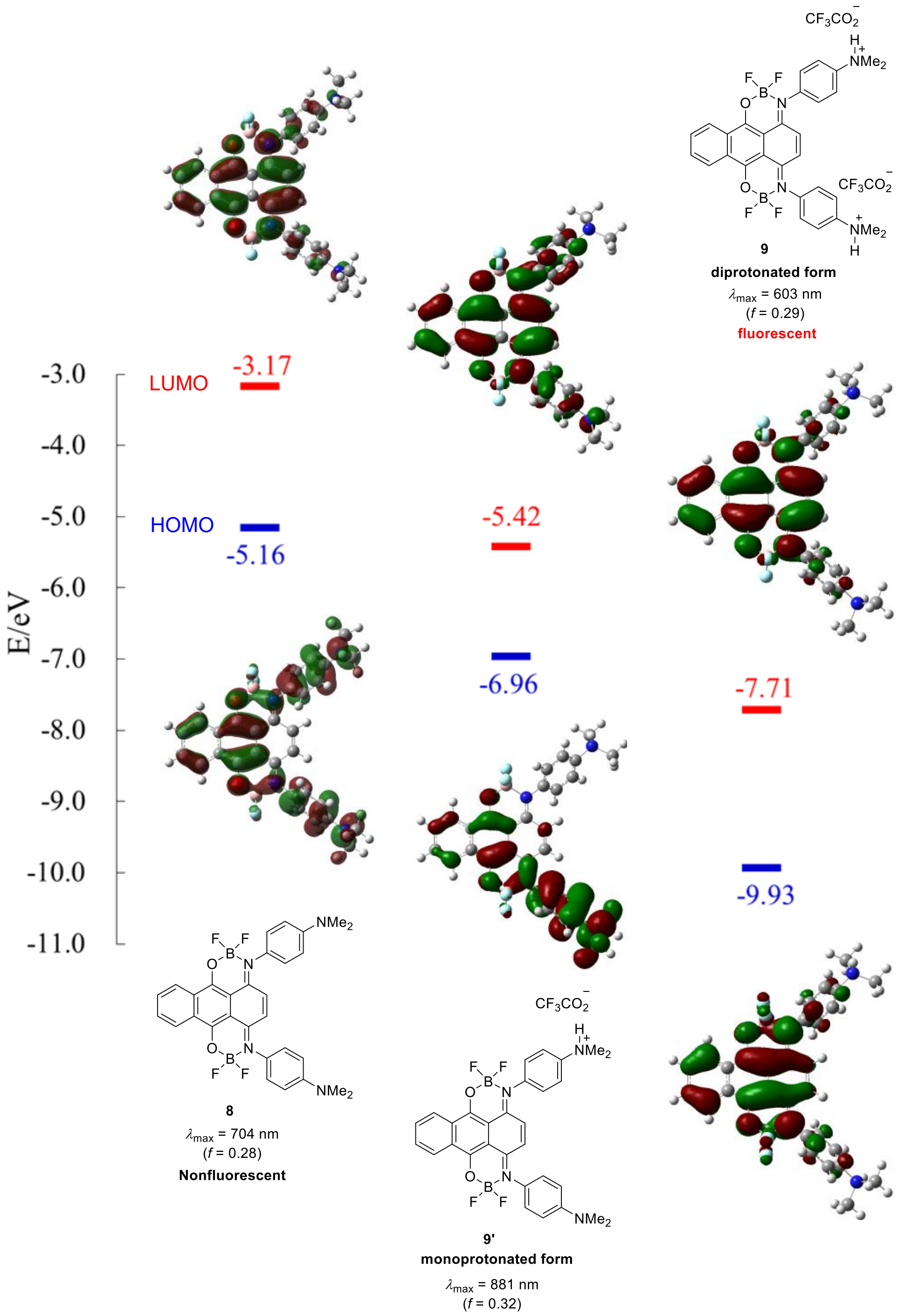

Figure S9. Protonation of dimethylamino-substituted 1,4-diboron complex **8**. The maximum absorption

wavelengths (λ_{max}) and oscillator strengths (*f*) were calculated by DFT and TDDFT at the B3LYP/6-31G(d,p) level. Calculations for the monoprotonated form **9'** and the diprotonated form **9** were performed without counteranions.

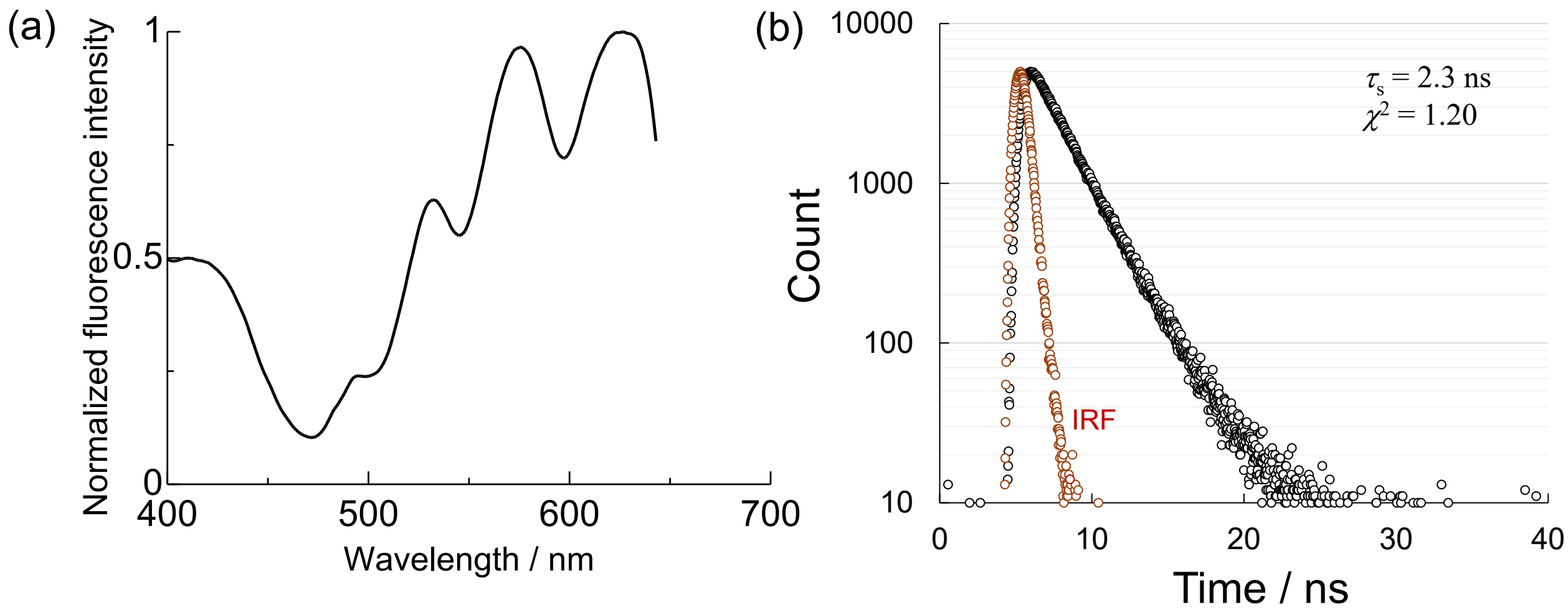

Figure S10. (a) Excitation spectrum and (b) fluorescence decay of **8** upon addition of 1000 equivalents of TFA. Time-resolved fluorescence measurements were performed at an excitation wavelength of 470 nm. The fluorescence decay curves were analyzed by reconvolution fitting using the measured instrument response function (IRF).

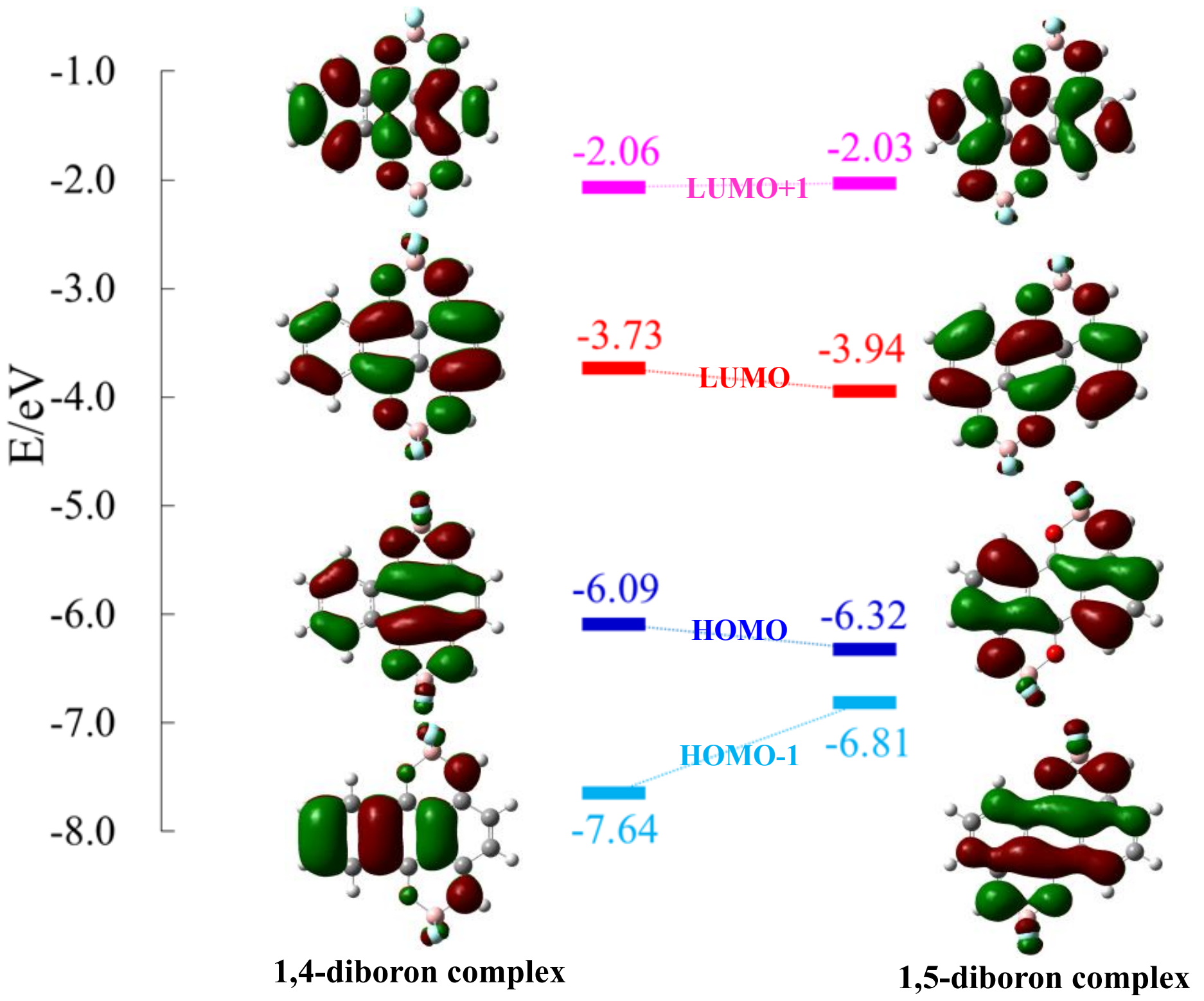

Figure S11. Molecular orbital energy diagrams and isodensity surface plots of the isolated acceptor moieties.

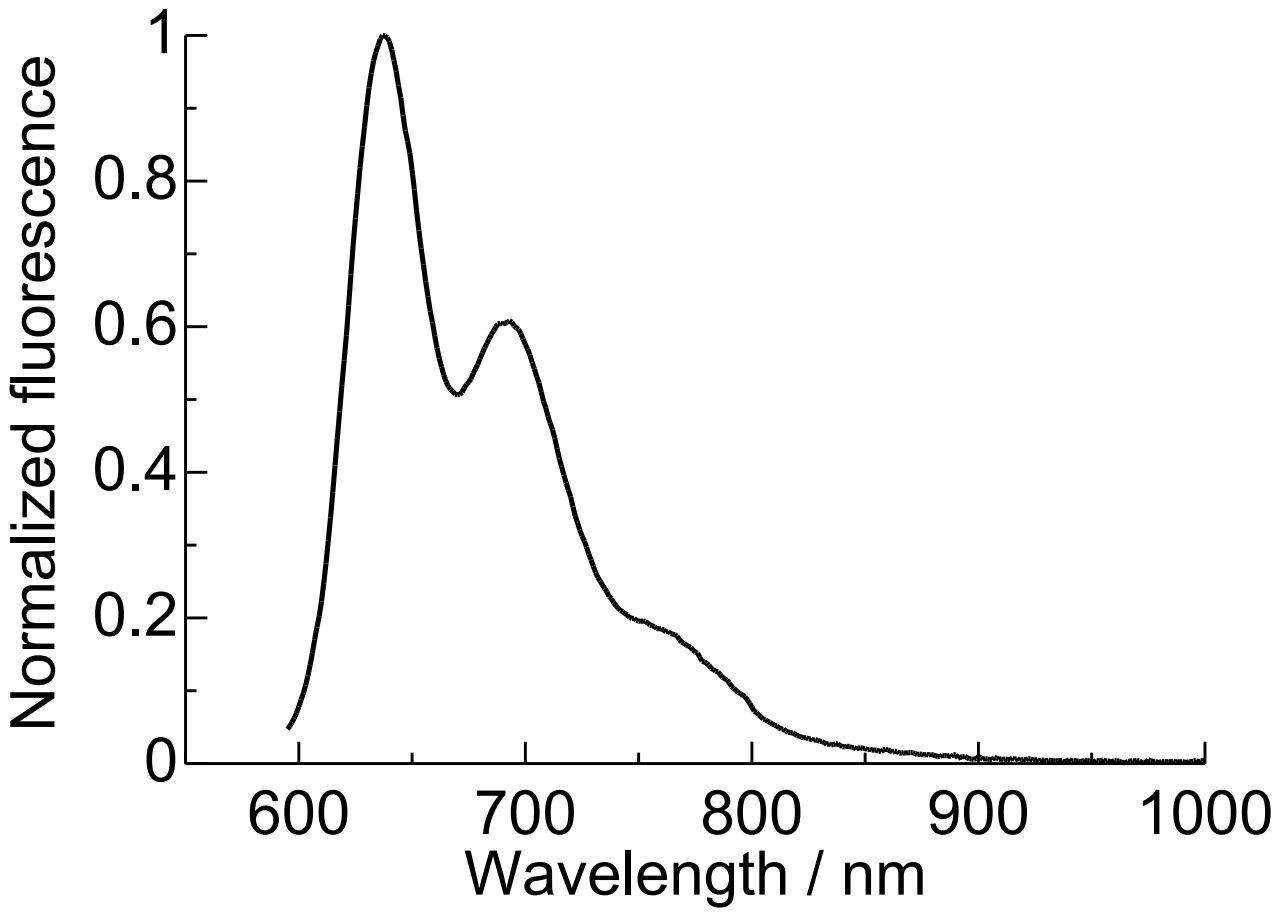

Figure S12. Normalized fluorescence spectra of **5** in aqueous formulated solution.

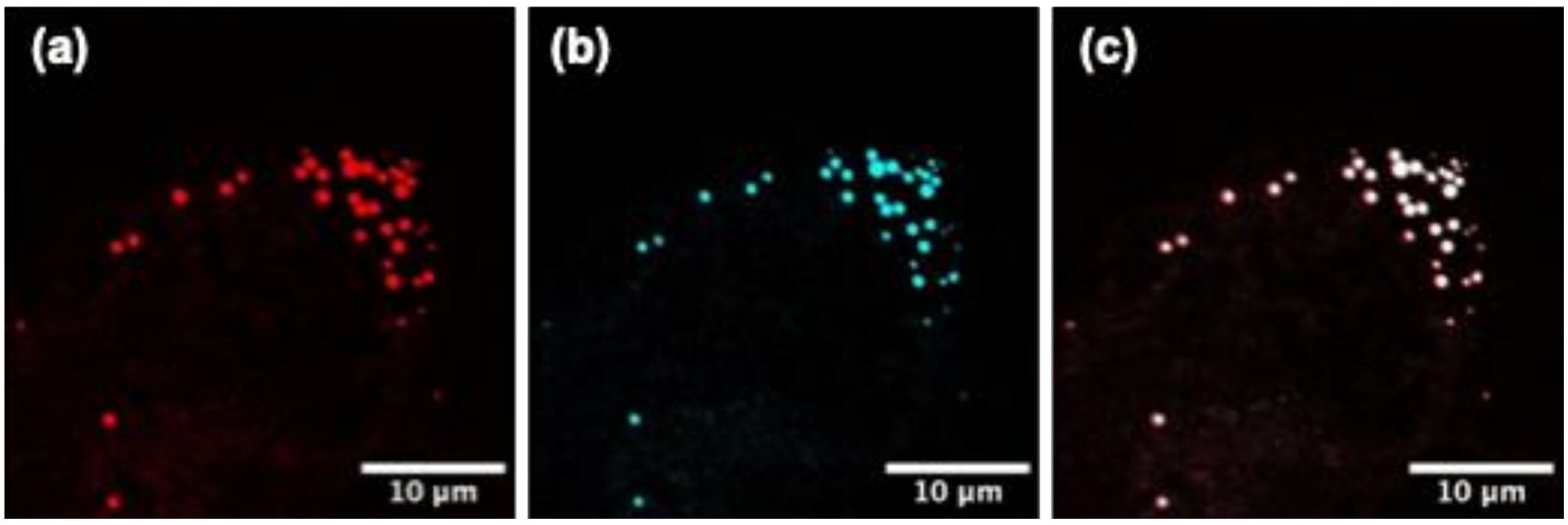

Figure S13. Confocal fluorescence images of live HeLa cells co-stained with **5** (500 nM) and LipiDye II (1 μM). Fluorescence images of (a) **5** and (b) LipiDye II. (c) Merged image. Acquisition conditions: for **5**, λ_{ex} = 561 nm and λ_{em} = 600–700 nm; for LipiDye II, λ_{ex} = 405 nm, λ_{em} = 490–550 nm. Scale bar: 10 μm.

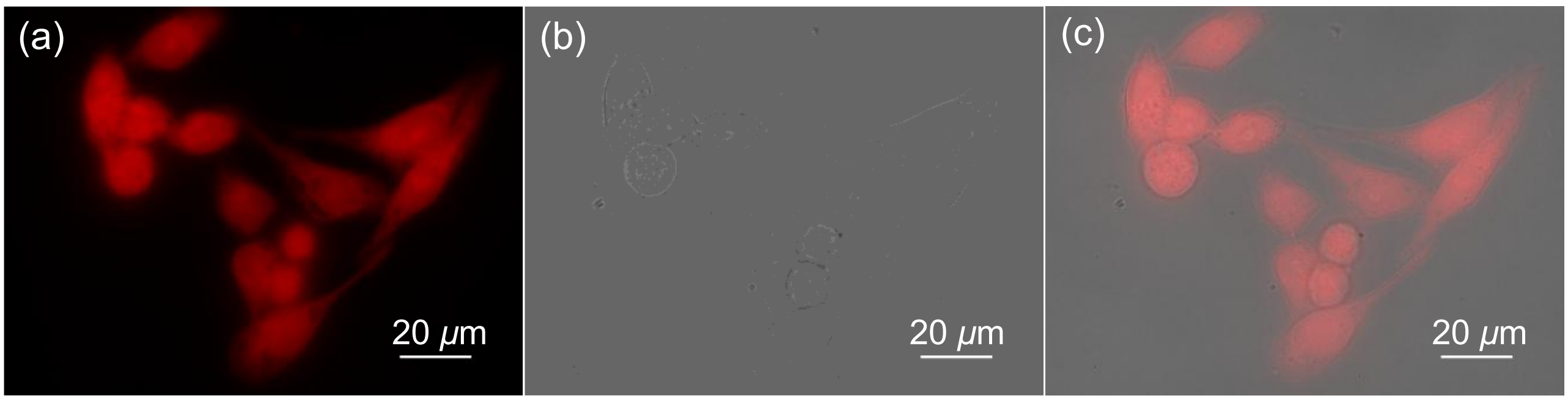

Figure S14. (a) Widefield fluorescence images of fixed HeLa cells stained with 10 μM of **5** for 1 h, acquired using LED excitation at 540-580 nm (bandpass filter 560/40), a dichroic mirror at 585 nm, and an emission filter at 630/75 nm. (b) bright-field image. (c) merged image.

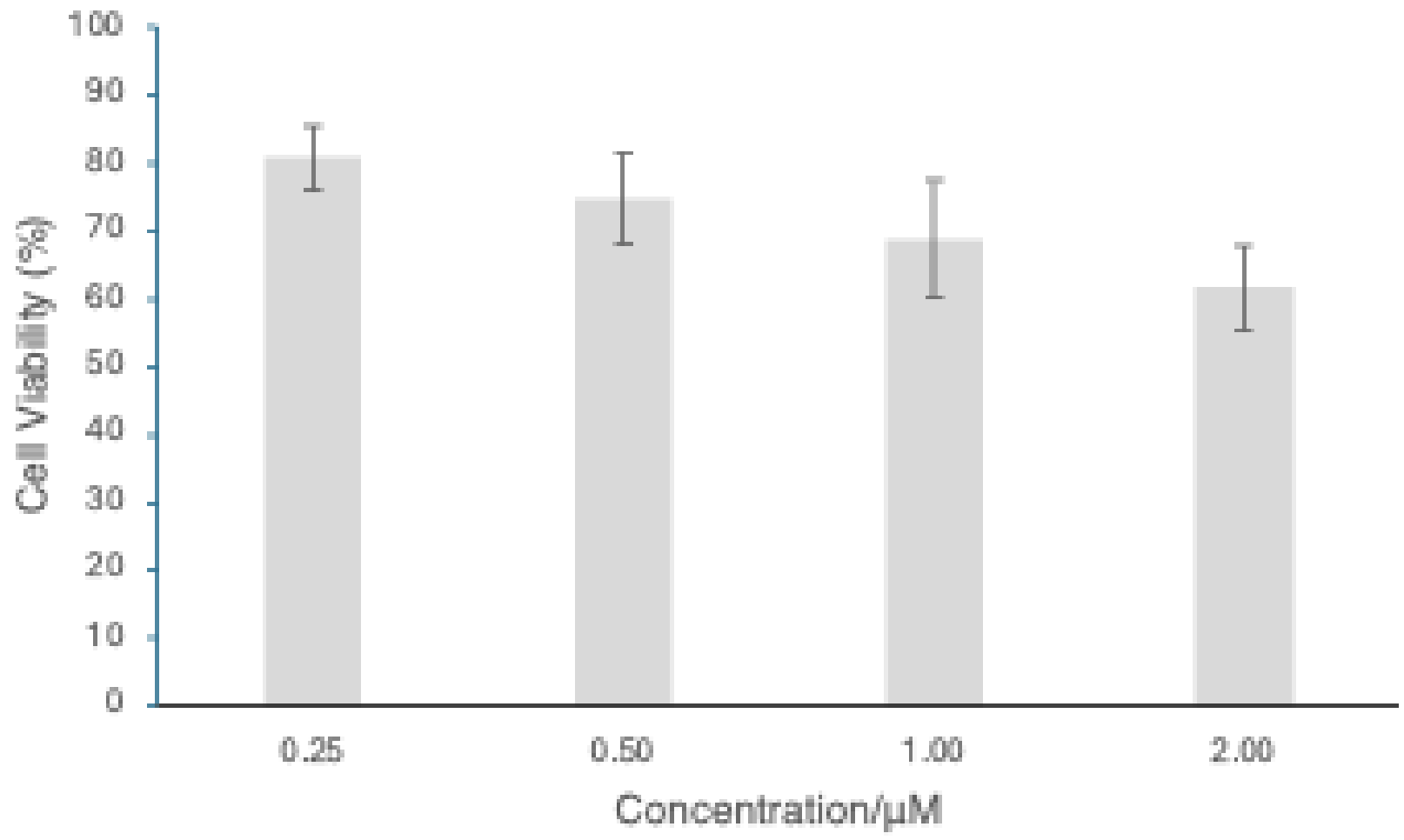

Figure S15. Cell viability of HeLa cells after 24 h incubation with **5** determined by the MTT assay. Results are expressed as percentages relative to the dye-free control. Error bars represent standard deviation (*N* = 12).

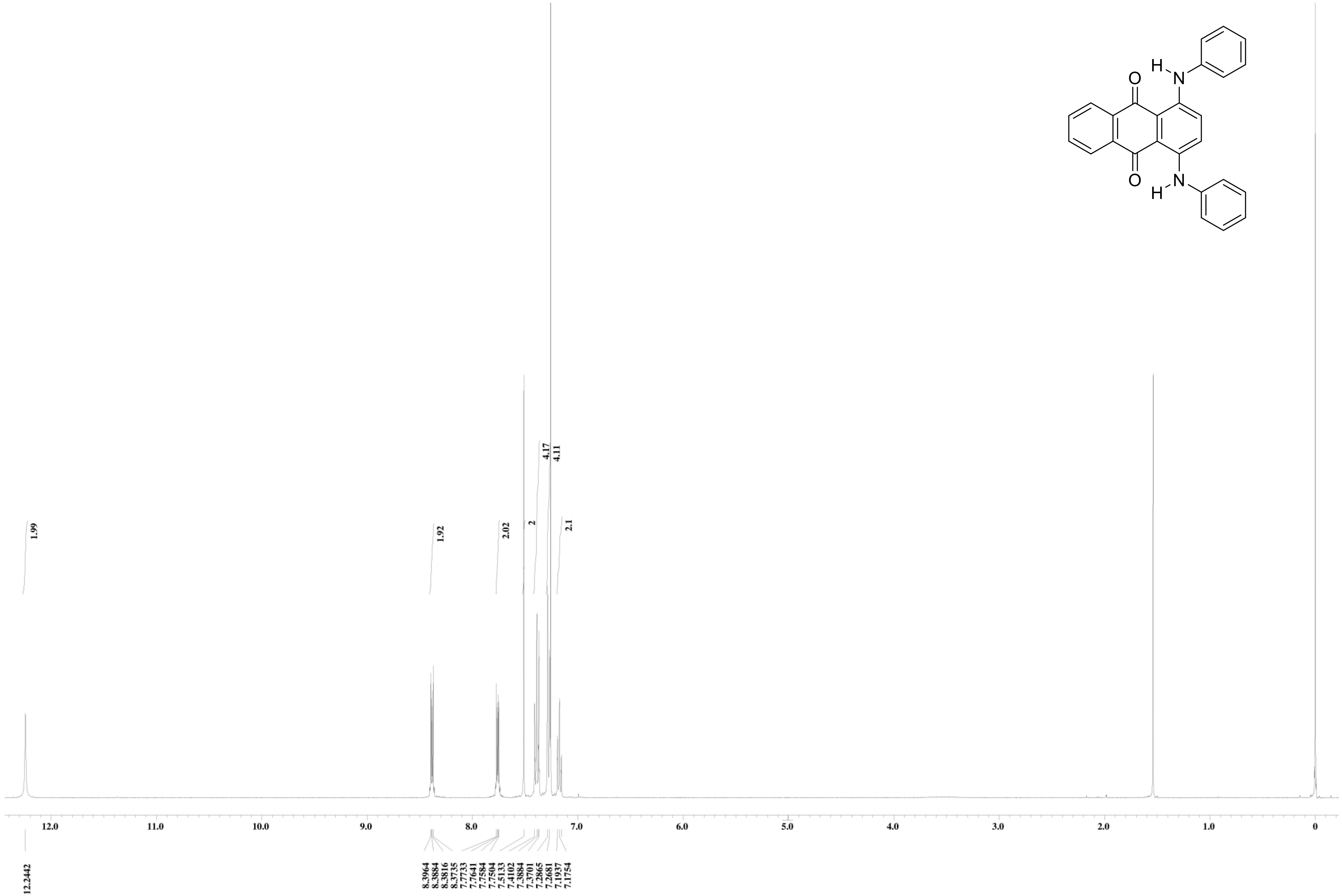

Figure S16. ^{1}H NMR spectrum of **1** (400 MHz, $CDCl_3$).

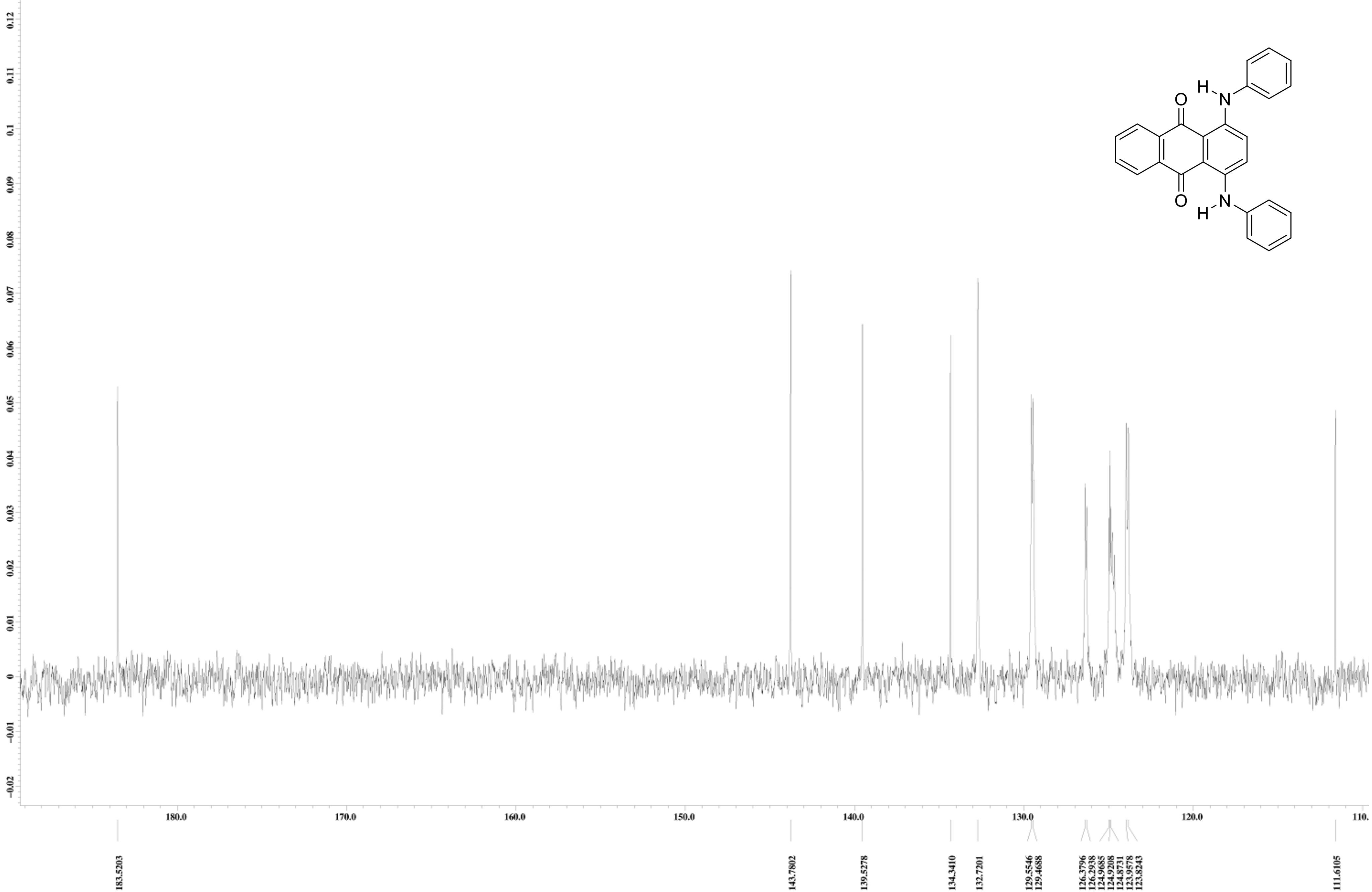

Figure S17. ^{13}C NMR spectrum of **1** (100 MHz, $CDCl_3$).

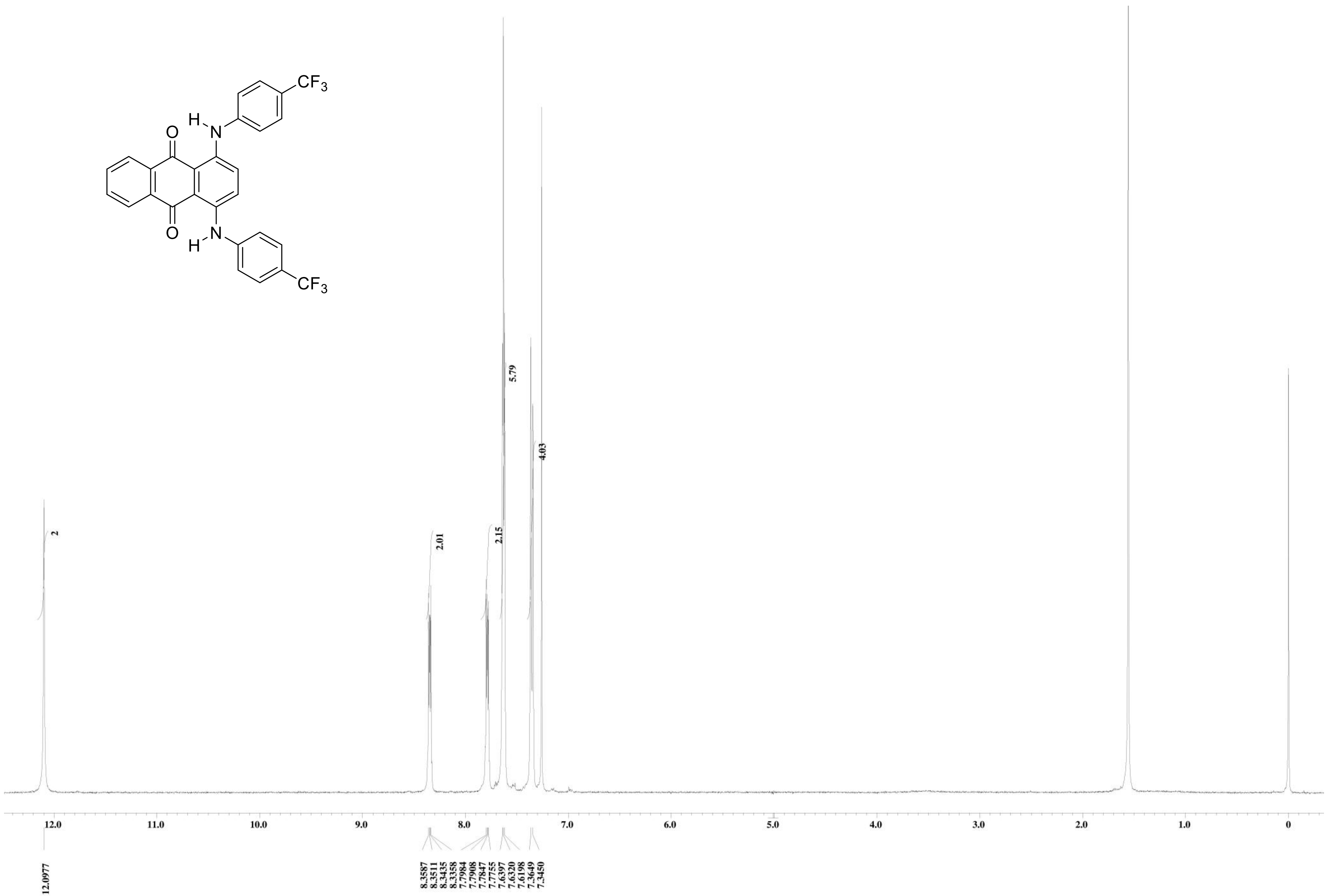

Figure S18. ^{1}H NMR spectrum of **2** (400 MHz, $CDCl_3$).

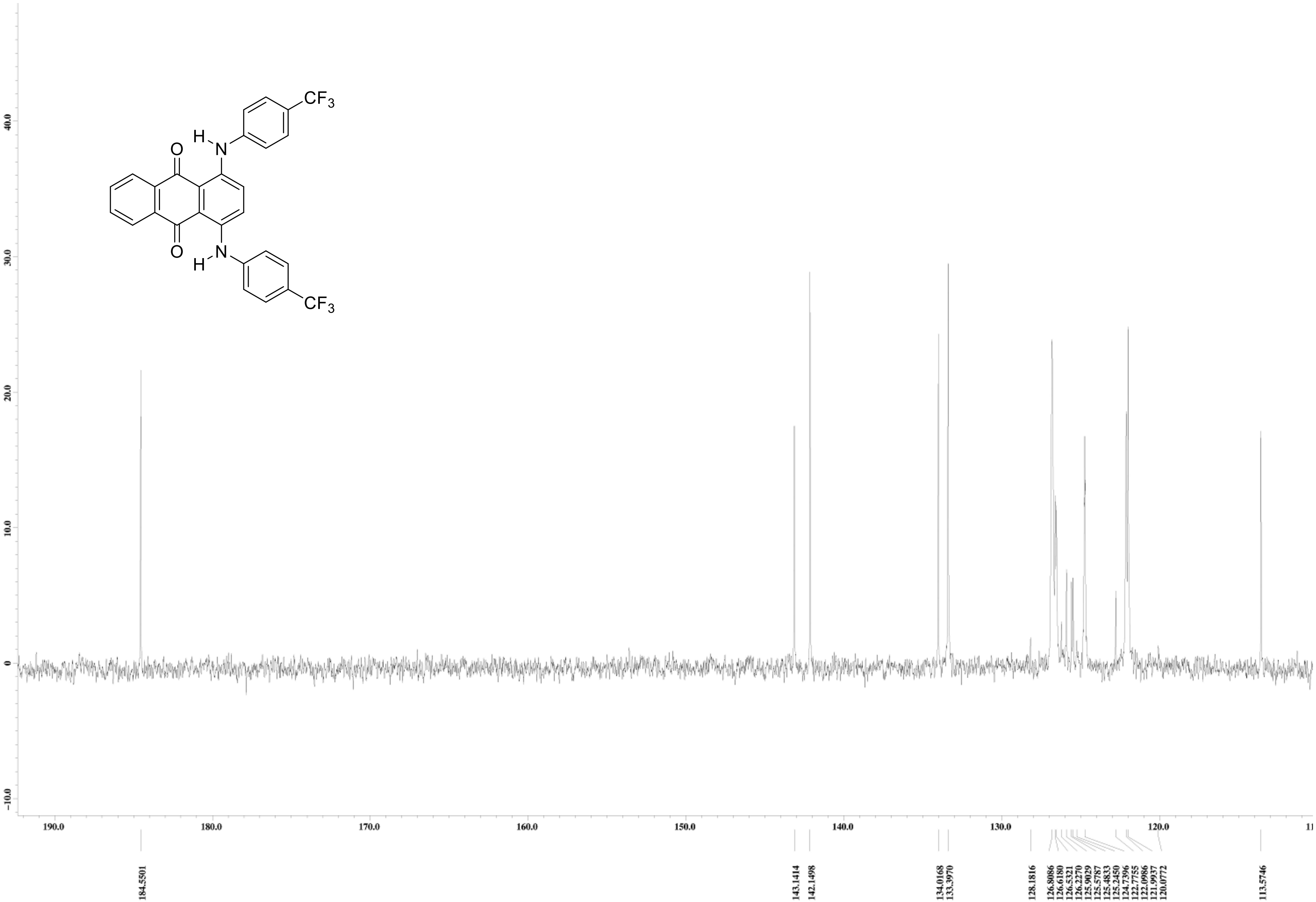

Figure S19. ^{13}C NMR spectrum of **2** (100 MHz, $CDCl_3$).

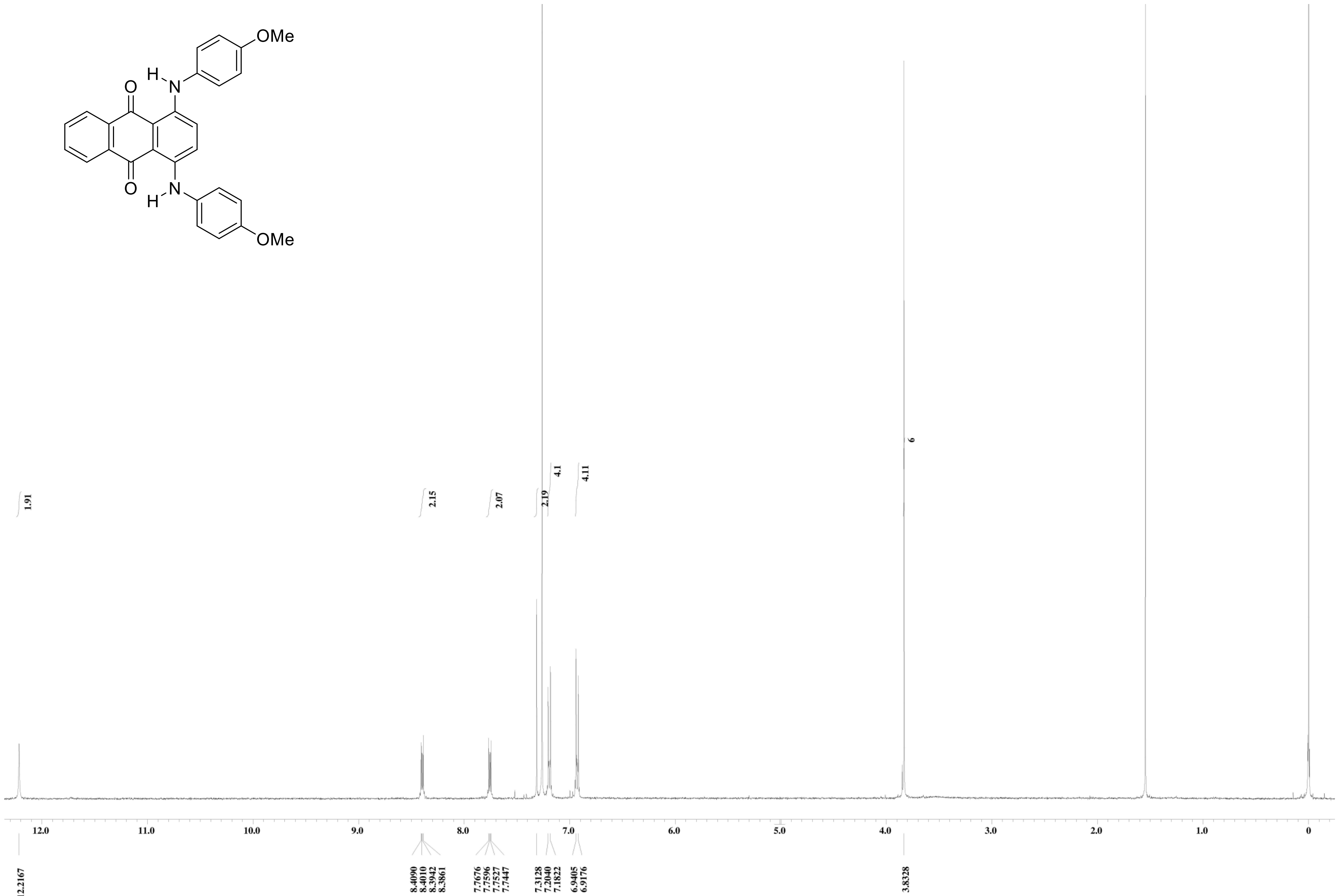

Figure S20. ^{1}H NMR spectrum of **3** (400 MHz, $CDCl_3$).

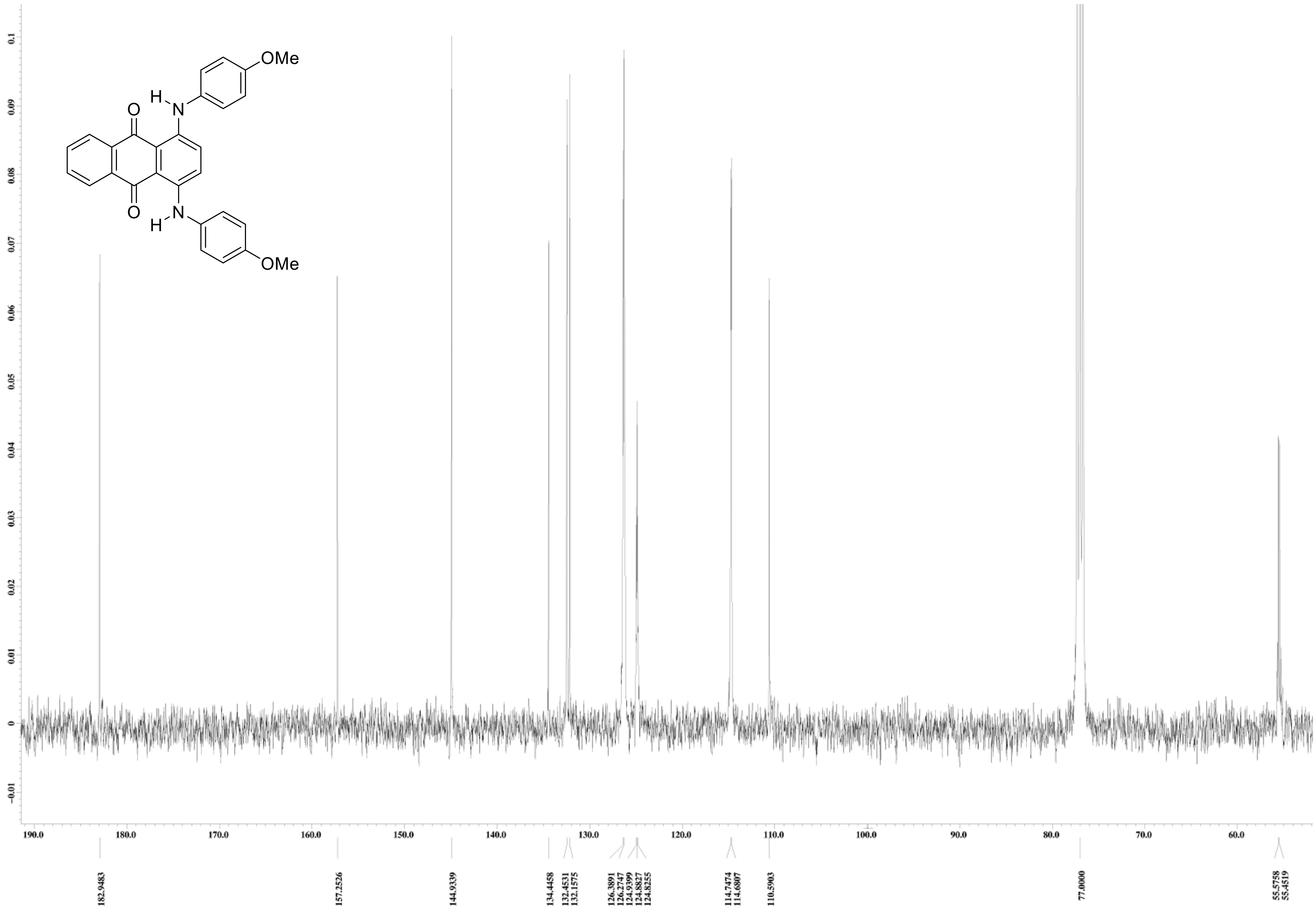

Figure S21. ^{13}C NMR spectrum of **3** (100 MHz, $CDCl_3$).

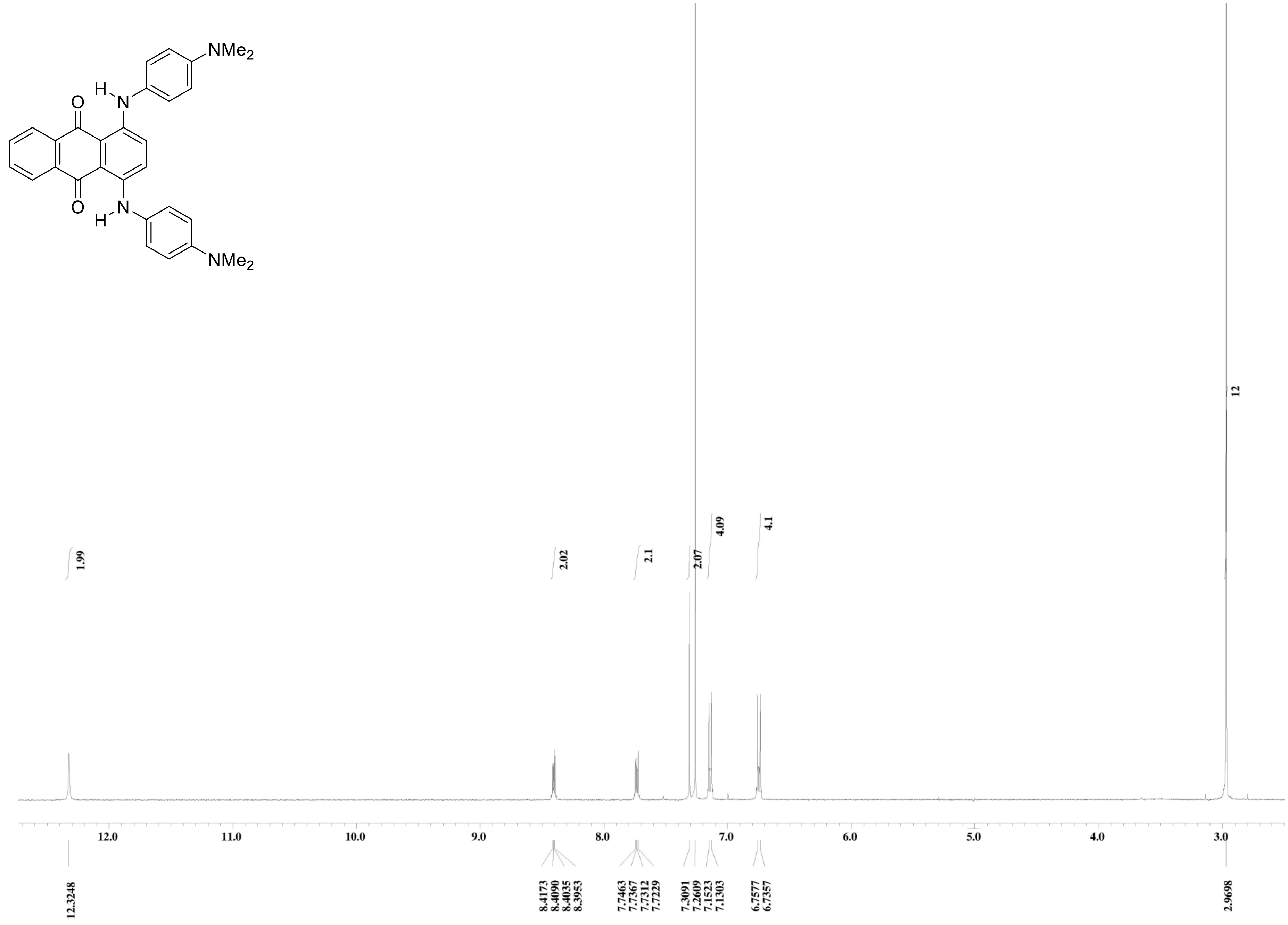

Figure S22. ^{1}H NMR spectrum of **4** (400 MHz, $CDCl_3$).

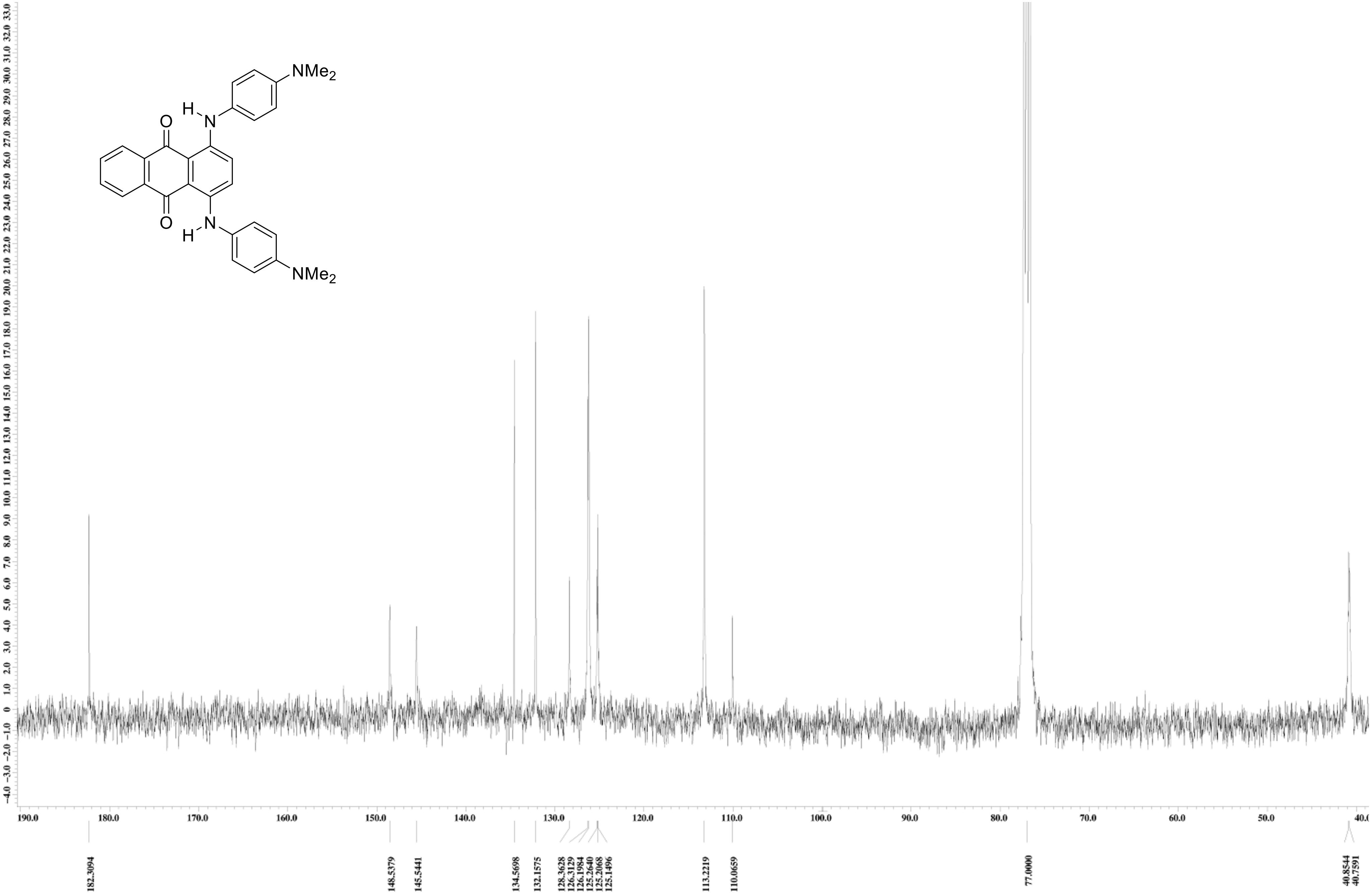

Figure S23. ^{13}C NMR spectrum of **4** (100 MHz, $CDCl_3$).

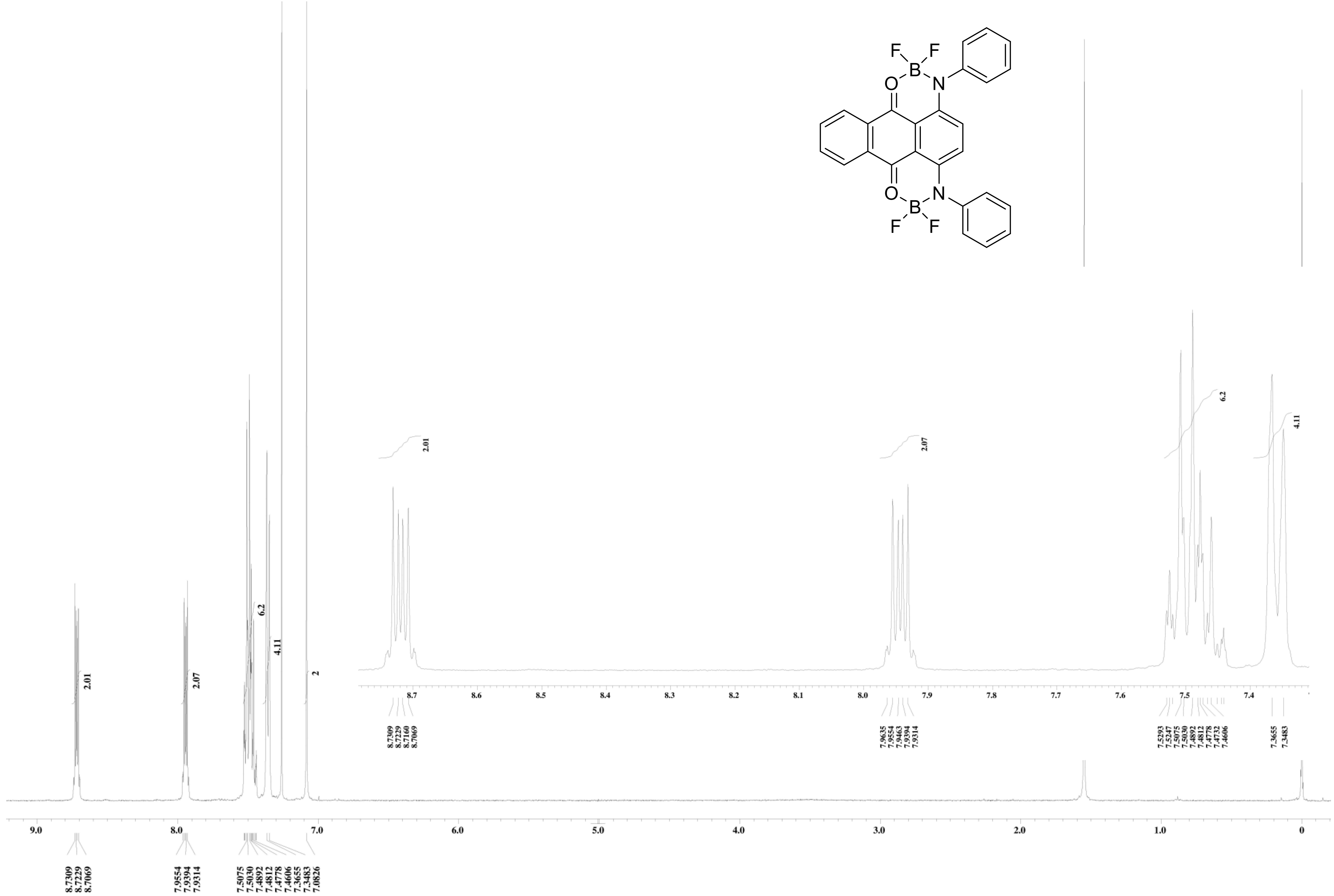

Figure S24. ^{1}H NMR spectrum of **5** (400 MHz, CDCl$_3$).

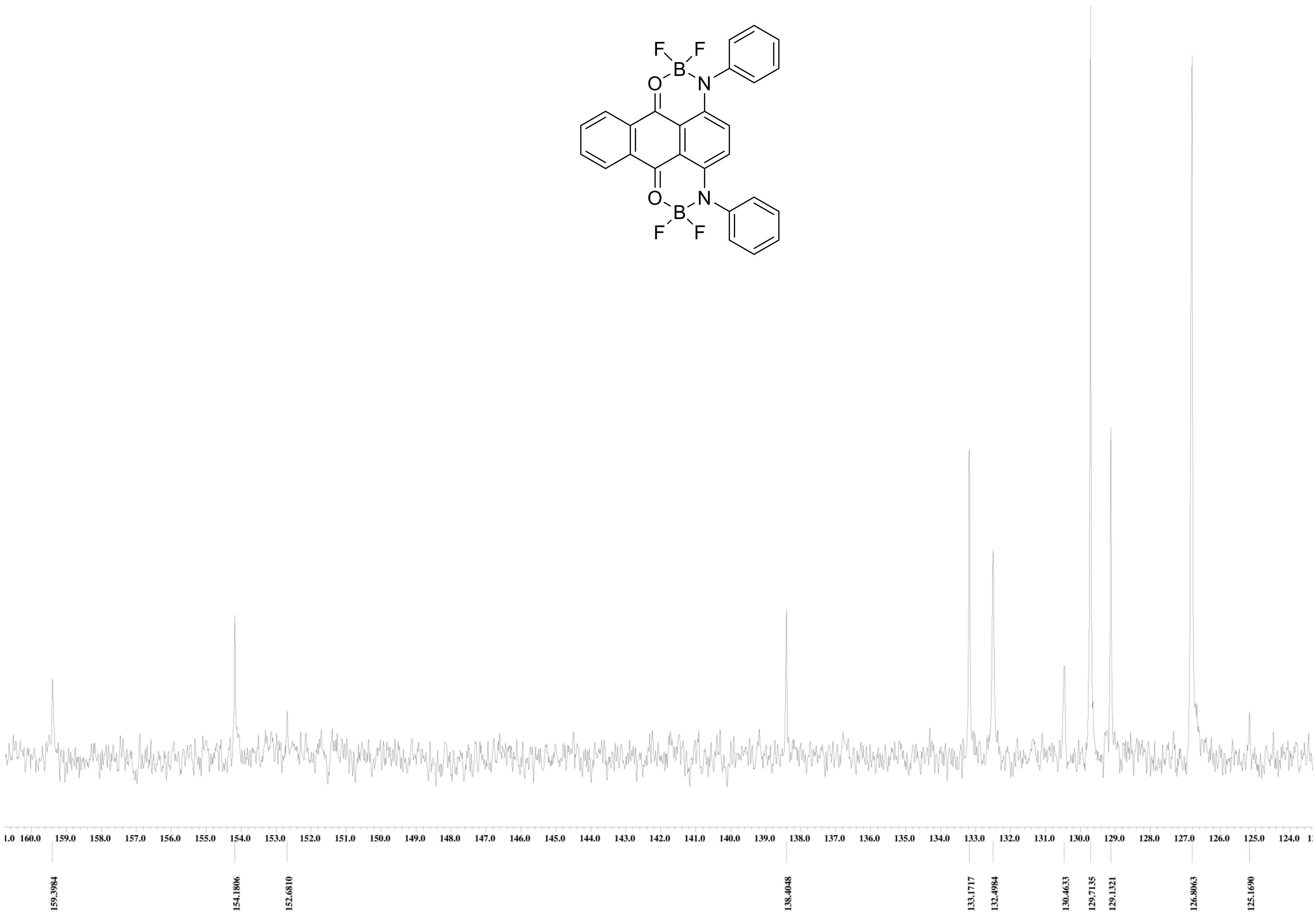

Figure S25. ^{13}C NMR spectrum of **5** (100 MHz, $CDCl_3$).

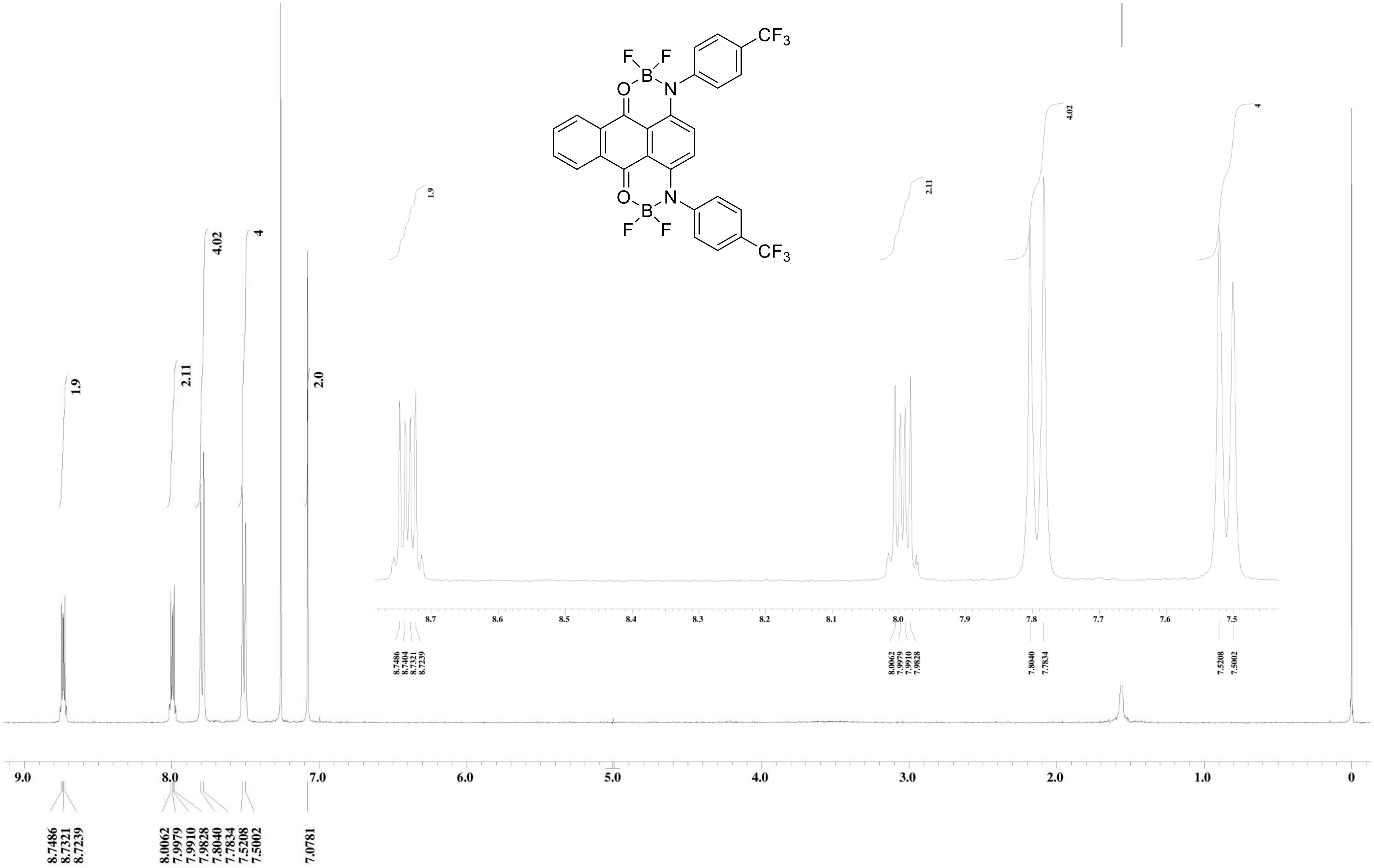

Figure S26. ^{1}H NMR spectrum of **6** (400 MHz, $CDCl_3$).

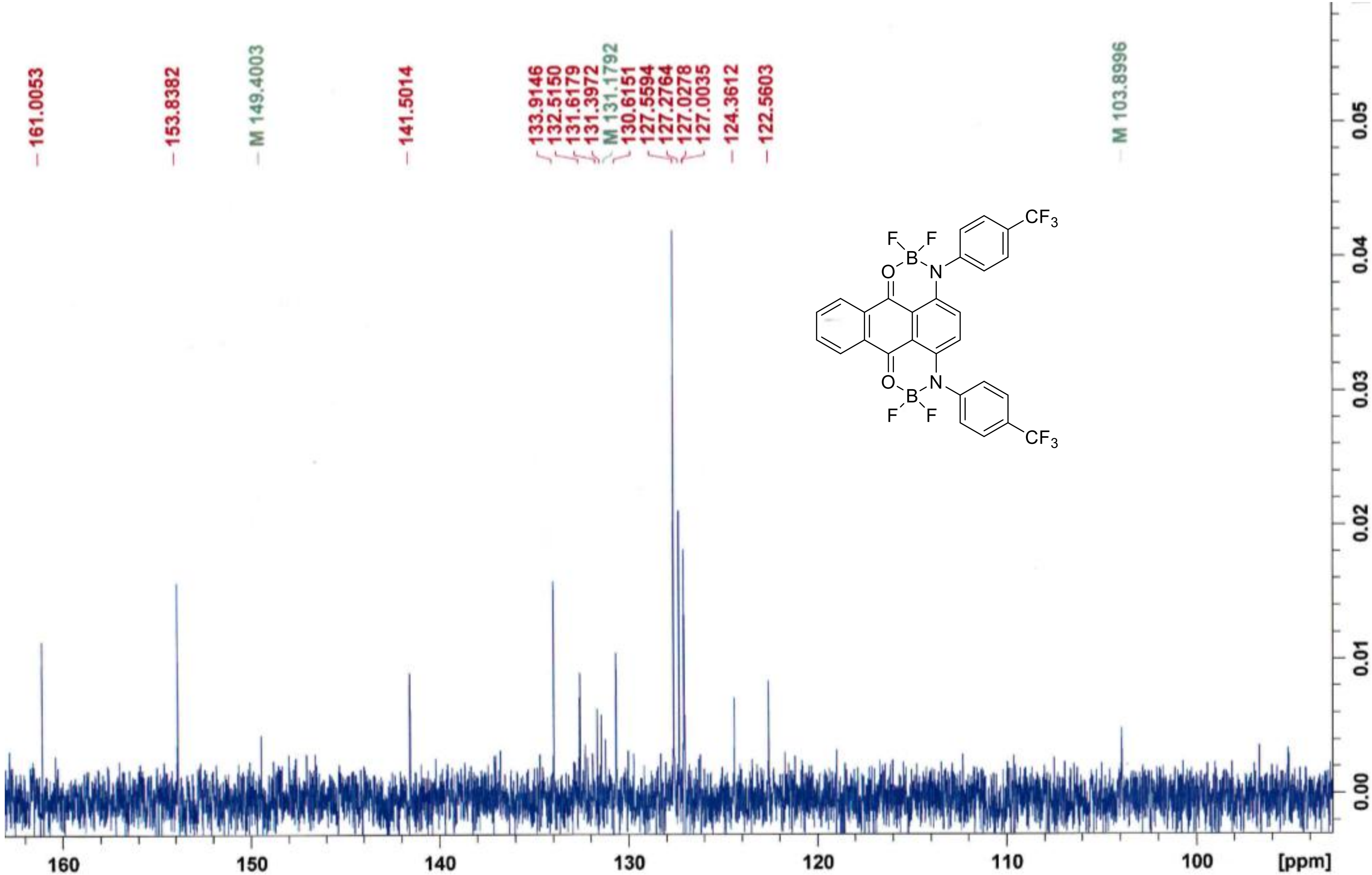

Figure S27. ^{13}C NMR spectrum of **6** (150 MHz, $CDCl_3$).

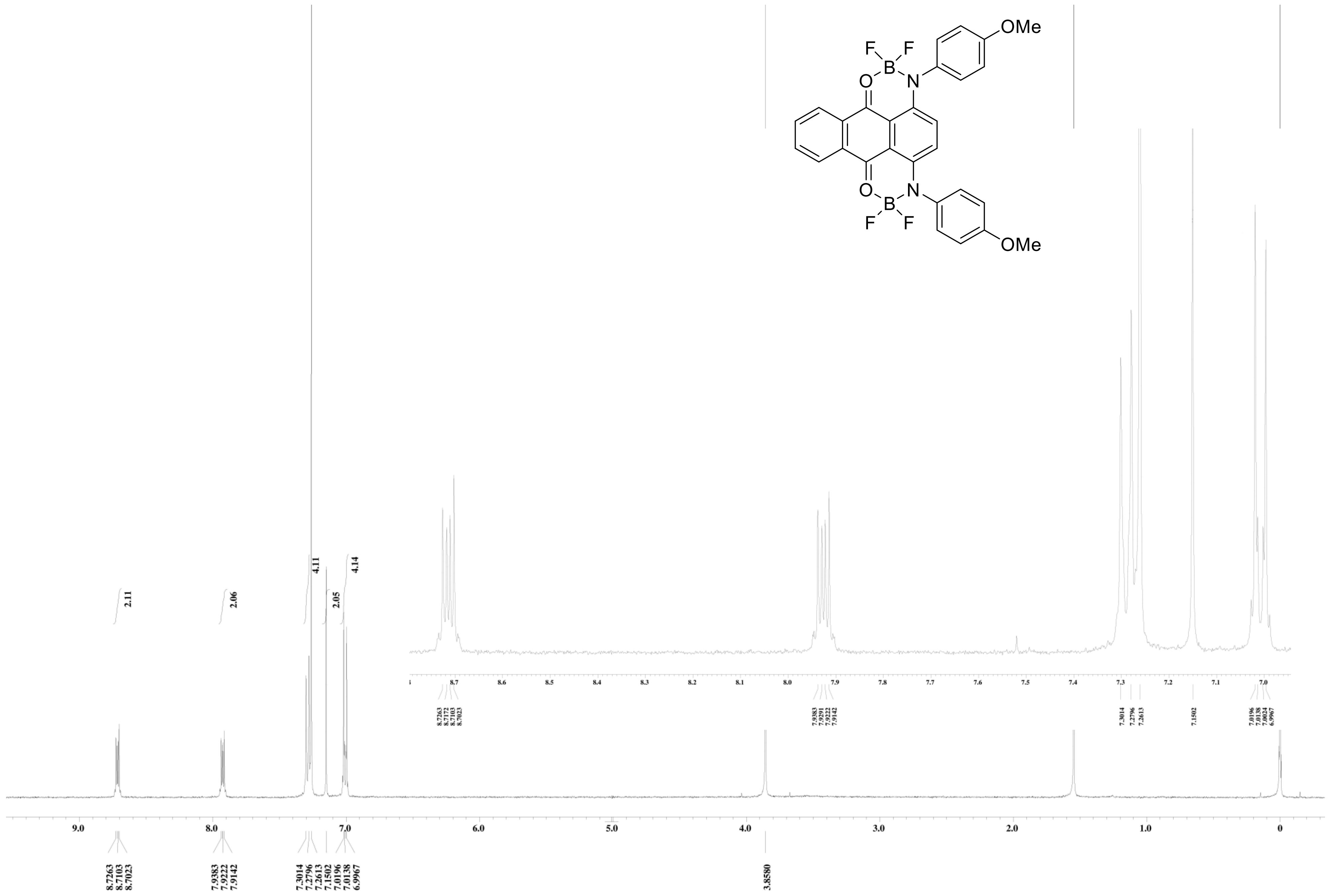

Figure S28. ^{1}H NMR spectrum of **7** (400 MHz, $CDCl_3$).

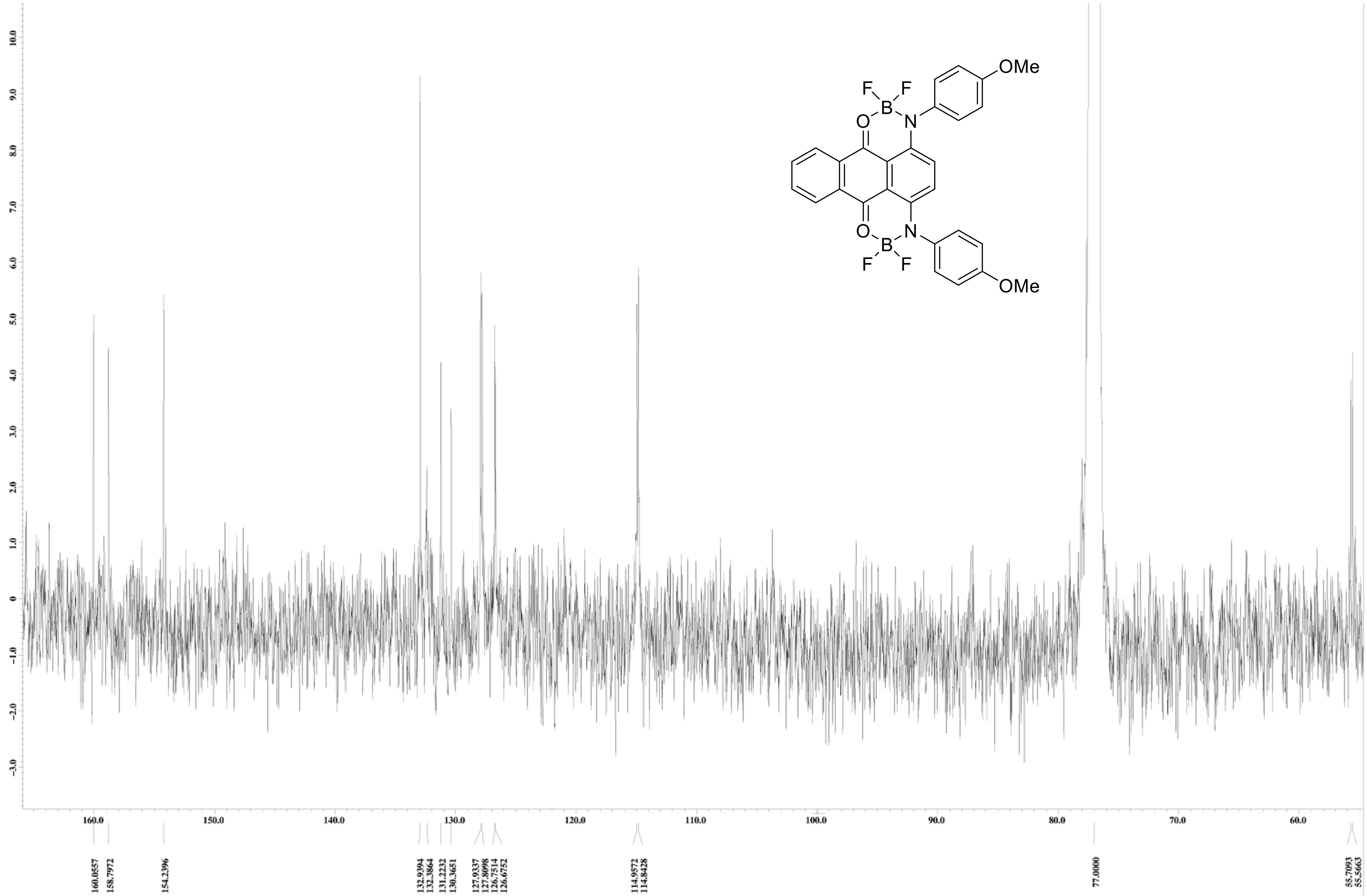

Figure S29. ^{13}C NMR spectrum of **7** (100 MHz, $CDCl_3$).

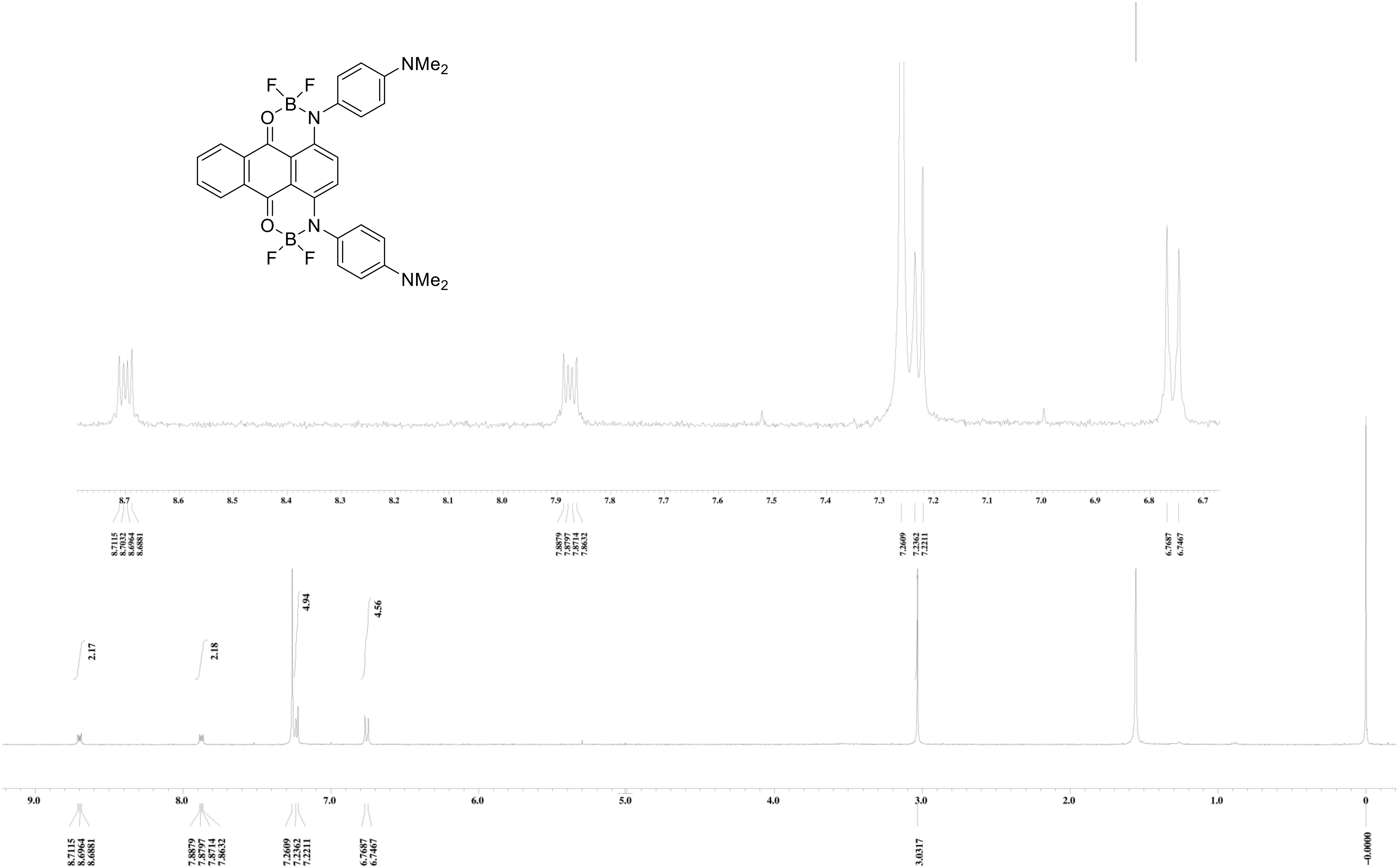

Figure S30. ^{1}H NMR spectrum of **8** (400 MHz, $CDCl_3$).

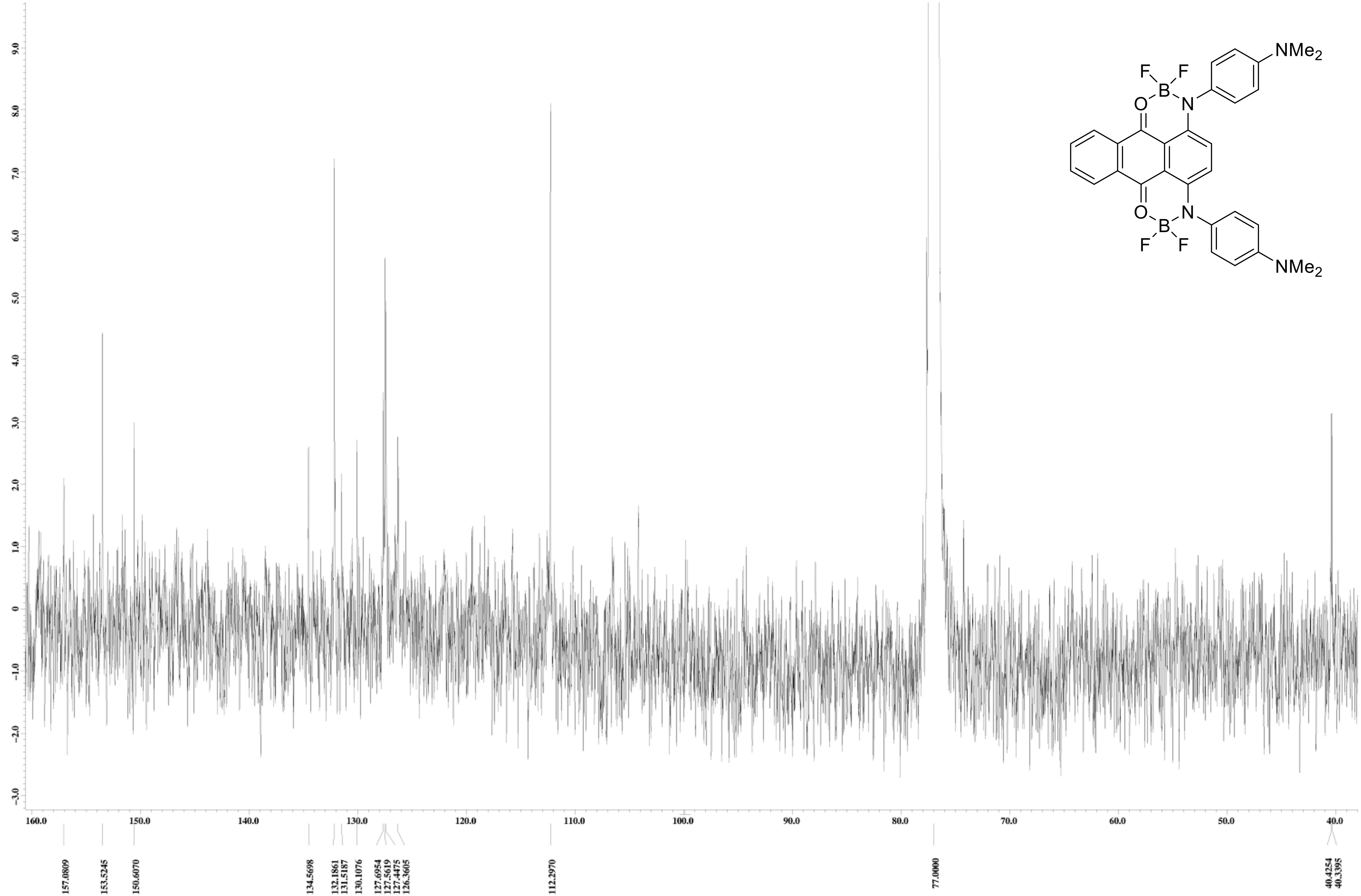

Figure S31. ^{13}C NMR spectrum of **8** (100 MHz, $CDCl_3$).
