# Supplementary material for: Synthesis and Optical Properties of Diboron Complexes of 1,4‐Anthraquinone Derivatives
Source: Chemistry. 2026 May 25;32(34):e71179. doi: 10.1002/chem.71179 (PMC13569954; doi:10.1002/chem.71179)

checkCIF/PLATON report

Structure factors have been supplied for datablock(s) CrystalClear

THIS REPORT IS FOR GUIDANCE ONLY. IF USED AS PART OF A REVIEW PROCEDURE FOR PUBLICATION, IT SHOULD NOT REPLACE THE EXPERTISE OF AN EXPERIENCED CRYSTALLOGRAPHIC REFEREE.

No syntax errors found. CIF dictionary Interpreting this report

Datablock: CrystalClear

Bond precision:	C-C = 0.0039 A		Wavelength=0.71075
Cell:	a=13.964(3)	b=9.580(2)	c=17.066(5)
	alpha=90	beta=101.196(10)	gamma=90
Temperature:	293 K		

	Calculated	Reported
Volume	2239.6(9)	2239.6(9)
Space group	P 21/n	P 21/n
Hall group	-P 2yn	-P 2yn
Moiety formula	C26 H16 B2 F4 N2 O2	?
Sum formula	C26 H16 B2 F4 N2 O2	C26 H16 B2 F4 N2 O2
Mr	486.03	486.03
Dx,g cm-3	1.441	1.441
Z	4	4
Mu (mm-1)	0.113	0.113
F000	992.0	992.0
F000'	992.60	
h,k,lmax	18,12,22	18,12,22
Nref	5190	5003
Tmin,Tmax	0.978,0.978	
Tmin'	0.978	

Correction method= Not given

Data completeness= 0.964 Theta(max)= 27.594

R(reflections)= 0.0595(3045) wR2(reflections)= 0.1595(5003)

S = 1.027 Npar= 325

The following ALERTS were generated. Each ALERT has the format
test-name_ALERT_alert-type_alert-level.
Click on the hyperlinks for more details of the test.

Alert level C

```
PLAT029_ALERT_3_C _diffrn_measured_fraction_theta_full value Low .      0.971 Why?
PLAT052_ALERT_1_C Info on Absorption Correction Method   Not Given     Please Do !
PLAT241_ALERT_2_C High   'MainMol' Ueq as Compared to Neighbors of        C16 Check
PLAT241_ALERT_2_C High   'MainMol' Ueq as Compared to Neighbors of        C17 Check
PLAT241_ALERT_2_C High   'MainMol' Ueq as Compared to Neighbors of        C19 Check
PLAT242_ALERT_2_C Low    'MainMol' Ueq as Compared to Neighbors of        C15 Check
PLAT242_ALERT_2_C Low    'MainMol' Ueq as Compared to Neighbors of        C18 Check
PLAT331_ALERT_2_C Small Aver Phenyl C-C Dist C15      --C20      .       1.36 Ang.
PLAT906_ALERT_3_C Large K Value in the Analysis of Variance ......     24.611 Check
PLAT906_ALERT_3_C Large K Value in the Analysis of Variance ......      3.619 Check
PLAT910_ALERT_3_C Missing # of FCF Reflection(s) Below Theta(Min).          6 Note
                 1  1  0,  -1  0  1,  -1  1  1,   0  1  1,   1  0  1,   0  0  2,
PLAT911_ALERT_3_C Missing FCF Refl Between Thmin & STh/L=    0.600        113 Report
                 5  4  0,   8  7  0,   9  2  0,   9  8  0,  10  2  0,  10  7  0,
                11  2  0,  11  4  0,  12  1  0,  15  4  0, -11  2  1, -11  8  1,
                -9  5  1,  -8  1  1,  -4  3  1,   2  2  1,   4  9  1,   7  2  1,
                 7  4  1,  10  2  1,  15  3  1,  -7  6  2,  -7  7  2,  -6  1  2,
                -5  4  2,  -4  4  2,  -3 10  2,   7  2  2,  10  3  2,  10  7  2,
                12  6  2, -16  1  3, -10  1  3,   1  1  3,   4  3  3,   6 10  3,
                14  1  3, -13  4  4,  -9  6  4,  -6  3  4,   0  0  4,   2  2  4,
                 5  3  4, -12  3  5,  -9  6  5,  -8  6  5,  -4  3  5,  -3  8  5,
                -2 10  5,  -1  0  5,   0 11  5,  12  1  5,  14  1  5,  -6  2  6,
                 0  8  6,   9  2  6,  14  3  6,  -1  8  7,   3  7  7,   4  2  7,
                 6  4  7,   9  2  7, -10  8  8,  -9  5  8,  -6  7  8,  -2 10  8,
                 5  6  8,  10  7  8,  -8  1  9,  -4  5  9,  -3  1  9, -14  2 10,
                -9  5 10,  -6  9 10,  -5  4 10,   2  6 10,   4  9 10,   7  5 10,
               -13  3 11, -10  5 11,  -1  4 11,   1  1 11, -15  1 12,  -4  9 12,
                 2  6 12,   7  2 12,  -8  3 13,  -1  1 13,   2  1 13,   7  1 13,
                -8  1 14,  -4  6 14,  -3  3 14,  -3  7 14,  -1  7 14,   7  1 14,
```

Alert level G

```
PLAT199_ALERT_1_G Reported _cell_measurement_temperature ..... (K)         293 Check
PLAT200_ALERT_1_G Reported   _diffrn_ambient_temperature ..... (K)         293 Check
PLAT432_ALERT_2_G Short Inter X...Y Contact  F3       ..C1       .       2.82 Ang.
                                      1/2-x,-1/2+y,3/2-z  =      2_546 Check
PLAT883_ALERT_1_G Absent Datum for _atom_sites_solution_primary ..     Please Do !
PLAT912_ALERT_4_G Missing # of FCF Reflections Above STh/L=  0.600         67 Note
PLAT933_ALERT_2_G Number of HKL-OMIT Records in Embedded .res File        150 Note
                -1  0  1,  -5  4 10,   2  2  4,   9  2  0,   2  6 10,   0  1  1,
                -6  2  6,   5  6  8,  10  3 14,   4  9 10,  -6  3  4,   9  2  7,
               -10  1  3,  -8  1 14,  -3  8  5,  14  1  3,   4 10 10,   5  4  0,
                11  4  0,   6 10  3, -13  7  9, -12  8  5,  -4  9 12,  -7  1 15,
               -11  8  1,  13  7  3, -10  3 15,  10  2  0,  10  3  2,  14  3  6,
                 1  0  1,   1  2 18,  15  5  4, -13  8  6,  -1  4 11,  -9  5  8,
                -1  8  7,  12  6  2,  -1  2 20,  -3 10  2,  -9  6  4, -14  2 10,
                -5  1 20, -12  3  5,  -3  1  9,   7  2  1,   7  9 10, -10  8  8,
                 3  2 16,  -8  6  5,
PLAT941_ALERT_3_G Average HKL Measurement Multiplicity ..........         4.4 Low
PLAT969_ALERT_5_G The 'Henn et al.' R-Factor-gap value ..........       4.290 Note
```

```
              Predicted wR2: Based on SigI**2  3.72 or SHELX Weight 15.53
PLAT978_ALERT_2_G Number C-C Bonds with Positive Residual Density.         0 Info
PLAT992_ALERT_5_G Repd & Actual _reflns_number_gt Values Differ by         2 Check
```

```
   0 ALERT level A = Most likely a serious problem - resolve or explain
   0 ALERT level B = A potentially serious problem, consider carefully
  12 ALERT level C = Check. Ensure it is not caused by an omission or oversight
  10 ALERT level G = General information/check it is not something unexpected

   4 ALERT type 1 CIF construction/syntax error, inconsistent or missing data
   9 ALERT type 2 Indicator that the structure model may be wrong or deficient
   6 ALERT type 3 Indicator that the structure quality may be low
   1 ALERT type 4 Improvement, methodology, query or suggestion
   2 ALERT type 5 Informative message, check
```

It is advisable to attempt to resolve as many as possible of the alerts in all categories. Often the minor alerts point to easily fixed oversights, errors and omissions in your CIF or refinement strategy, so attention to these fine details can be worthwhile. In order to resolve some of the more serious problems it may be necessary to carry out additional measurements or structure refinements. However, the purpose of your study may justify the reported deviations and the more serious of these should normally be commented upon in the discussion or experimental section of a paper or in the "special_details" fields of the CIF. checkCIF was carefully designed to identify outliers and unusual parameters, but every test has its limitations and alerts that are not important in a particular case may appear. Conversely, the absence of alerts does not guarantee there are no aspects of the results needing attention. It is up to the individual to critically assess their own results and, if necessary, seek expert advice.

Publication of your CIF in IUCr journals

A basic structural check has been run on your CIF. These basic checks will be run on all CIFs submitted for publication in IUCr journals (*Acta Crystallographica*, *Journal of Applied Crystallography*, *Journal of Synchrotron Radiation*); however, if you intend to submit to *Acta Crystallographica Section C* or *E* or *IUCrData*, you should make sure that full publication checks are run on the final version of your CIF prior to submission.

Publication of your CIF in other journals

Please refer to the *Notes for Authors* of the relevant journal for any special instructions relating to CIF submission.

PLATON version of 19/12/2024; check.def file version of 19/12/2024

Datablock CrystalClear - ellipsoid plot

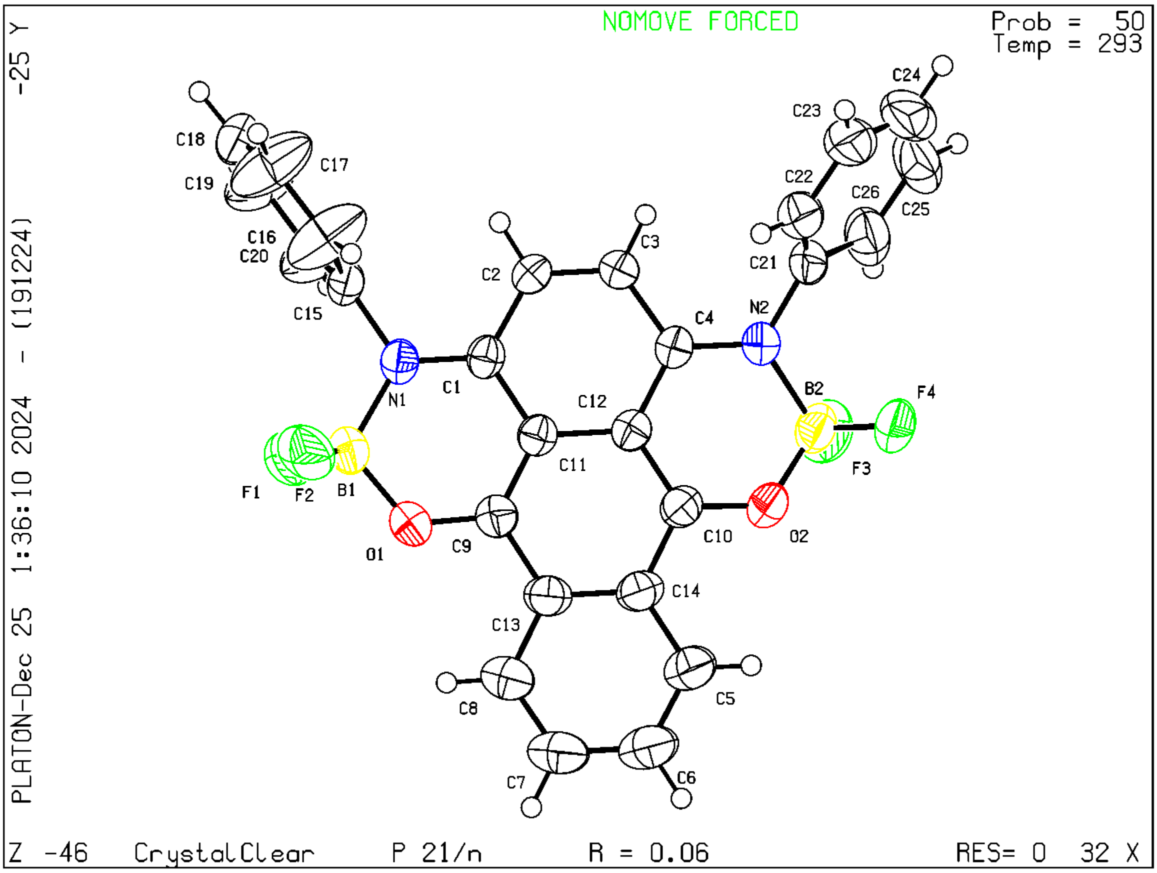

Supplement: Supplementary file 2 [file CHEM-32-e71179-s001.zip › chem71179-sup-0002-cif/checkcif_5_2.pdf]
